# Microarray analysis of gene expression in liver, adipose tissue and skeletal muscle in response to chronic dietary administration of NDGA to high-fructose fed dyslipidemic rats

**DOI:** 10.1186/s12986-016-0121-y

**Published:** 2016-09-29

**Authors:** Haiyan Zhang, Wen-Jun Shen, Yihang Li, Alex Bittner, Stefanie Bittner, Juveria Tabassum, Yuan F. Cortez, Fredric B. Kraemer, Salman Azhar

**Affiliations:** 1 Geriatric Research, Education and Clinical Center, VA Palo Alto Health Care System, Palo Alto, CA USA; 2 Division of Endocrinology, Stanford University, Stanford, CA USA; 3 Present Address: Department of Pathology, Keck School of Medicine, University of Southern California, Los Angeles, CA 90033 USA; 4 Present Address: Department of Pediatrics, Saint Louis University School of Medicine, St. Louis, MO USA

**Keywords:** Fatty acid oxidation, Lipogenesis, Steatosis, Hypertriglyceridemia, CD36, L-FABP, Malony-CoA decarboxylase

## Abstract

Nordihydroguaiaretic acid (NDGA), the main metabolite of Creosote Bush, has been shown to have profound effects on the core components of metabolic syndrome, including lowering of blood glucose, free fatty acids and triglyceride levels, attenuating elevated blood pressure in several rodent models of dyslipidemia, and improving body weight, insulin resistance, diabetes and hypertension. In the present study, a high-fructose diet fed rat model of hypertriglyceridemia, dyslipidemia, insulin resistance and hepatic steatosis was employed to investigate the global transcriptional changes in the lipid metabolizing pathways in three insulin sensitive tissues: liver, skeletal muscle and adipose tissue in response to chronic dietary administration of NDGA. Sprague-Dawley male rats (SD) were fed a chow (control) diet, high-fructose diet (HFrD) or HFrD supplemented with NDGA (2.5 g/kg diet) for eight weeks. Dietary administration of NDGA decreased plasma levels of TG, glucose, and insulin, and attenuated hepatic TG accumulation. DNA microarray expression profiling indicated that dietary administration of NDGA upregulated the expression of certain genes involved in fatty acid oxidation and their transcription regulator, PPARα, decreased the expression of a number of lipogenic genes and relevant transcription factors, and differentially impacted the genes of fatty acid transporters, acetyl CoA synthetases, elongases, fatty acid desaturases and lipid clearance proteins in liver, skeletal muscle and adipose tissues. These findings suggest that NDGA ameliorates hypertriglyceridemia and steatosis primarily by inhibiting lipogenesis and enhancing fatty acid catabolism in three major insulin responsive tissues by altering the expression of key enzyme genes and transcription factors involved in de novo lipogenesis and fatty acid oxidation.

## Background

Nonalcoholic fatty liver disease (NAFLD), the most common cause of chronic liver disease [1–3], is a major health problem worldwide [2, 4]. NAFLD encompasses a spectrum of disease ranging from simple steatosis (excessive accumulation of fat mainly in the form of triglycerides [5–7]), nonalcoholic steatohepatitis (NASH) that includes steatosis along with inflammation and fibrosis and end-stage disease, cirrhosis and liver failure [8, 9] and a subsequent potential for hepatocellular carcinoma [10]. During the past few decades, the prevalence and severity of NAFLD have been paralleled with that of obesity, type 2 diabetes and metabolic syndrome (MetS) [4, 11, 12] and many studies have reported a pathophysiologic association between NAFLD and these disorders [11–18]. MetS is the umbrella description given to a number of derangements including insulin resistance and glucose intolerance, (central) obesity, dyslipidemia, and hypertension [19–21]. NAFLD is closely tied to insulin resistance and hepatic dyslipidemia and is now considered as a hepatic manifestation of MetS [12, 14–17]. The current evidence indicates NAFLD is an independent risk factor for the development of type 2 diabetes [13, 17, 18] and cardiovascular disease [12, 22–24]. Currently, there are no designated therapies in the clinical management of NAFLD except lifestyle modification including weight reduction, diet and exercise, which are hard to comply with on a long-term schedule [25–27].

Although the exact cause of the rising prevalence of NAFLD is not fully understood, the rapid urbanization, over-nutrition (consumption of high-carbohydrate and high-fat calorie-rich diets), increasing consumption of sugar sweetened (such as sucrose [cane or beet sugar] and high-fructose corn syrup [HFCS]) caloric beverages (i.e., soft-drinks and colas) and increasingly sedentary lifestyles, all have been linked with the increasing incidence of this disease [28–33]. In recent years, considerable attention has been paid to the contribution of dietary carbohydrates, sugar-sweetened beverages and monosaccharide fructose, in particular, in the pathogenesis of NAFLD [28, 30, 32, 34, 35]. The role of fructose in NAFLD (steatosis) pathogenesis has received a great deal of attention in part because from 1999 to 2004 the consumption of fructose increased by ~32 % [36]. HFCS now represents nearly 50 % of caloric sweeteners used in the United States [37]. Hepatic fructose metabolism differs from glucose [35, 38], and, in a hypercaloric setting, fructose induces hypertriglyceridemia and visceral adiposity, promotes lipogenesis and ectopic lipid accumulation, and decreases insulin sensitivity in humans [30, 32, 39–44] as well as rodents [30, 38, 45–52]. Based on these various findings, it appears that excessive consumption of fructose contributes to NAFLD pathogenesis in two ways: 1) it promotes TG production via de novo lipogenesis resulting in hyperlipidemia; and 2) it contributes to inflammation resulting in insulin resistance, steatosis, hepatic inflammation and fibrosis [53].

The creosote bush – *Larrea tridentate* – which grows abundantly in the North American and Mexican deserts, has been used by tribes native to these areas in the treatment of a wide range of ailments, including kidney and gallbladder stones, infertility, chicken pox, tuberculosis, cancer, venereal disease, colds, pain, arthritis and inflammation [54]. Previous work from our laboratory ([55, 56] and references therein) and others [57, 58] has shown that nordihydroguaiaretic acid (NDGA), the main metabolite of Creosote Bush [54], has profound effects on the core components of metabolic syndrome, including lowering of blood glucose, free fatty acids (FFA) and triglyceride (TG) levels, and attenuating elevated blood pressure in several rodent models of dyslipidemia, as well as improving body weight (obesity), insulin resistance, diabetes and hypertension. In a recently published study [59] we provided evidence using control, high-fat diet fed C57BL/6 J pre-diabetes (diet-induced obesity [DIO]), ob/ob control and NDGA fed DIO and *ob/ob* mice that NDGA exerts its hypolipidemic actions, including amelioration of hepatic steatosis, predominantly by stimulating the activity of the nuclear hormone receptor, peroxisome proliferator activated α (PPARα or NR1C1), the master regulator of all three hepatic fatty acid oxidation systems [60–67], which, in turn, improves dyslipidemia by promoting increased channeling of fatty acids towards their oxidation, and thus, restricting very low-density lipoprotein (VLDL)-TG production, storage, and secretion. We additionally observed that PPARα-independent pathways might also contribute to NDGA’s action to ameliorate hepatic steatosis.

To gain further insight into the molecular mechanisms underlying NDGA attenuation of dyslipidemia, including hypertriglyceridemia and hepatic steatosis, we utilized a microarray approach, which allowed us to observe the global effect of NDGA on lipid metabolism in three insulin sensitive tissues, liver, skeletal muscle and adipose tissues from high-fructose diet (HFrD)-induced hypertensive, hyperinsulinemic and hypertriglyceridemic rats. More specifically, the major goal of this study was to examine the effects of NDGA on expression of genes involved in lipid metabolism using HFrD fed rats to identify expression changes that might explain metabolic (i.e., insulin resistance, steatosis and hypertriglyceridemia) and histological (i.e., lipid droplet density) that are observed with NDGA treatment. Our results indicate that NDGA affects the expression of a large number of genes involved in fatty acid catabolism and synthesis in liver, muscle, and adipose tissue. In addition, based on these two affected pathways, several genes have been identified that may mediate the anti-hyperlipidemic actions of NDGA.

## Results

### Effect of chronic dietary NDGA treatment on physical and metabolic characteristics of high-fructose diet fed rats

We examined the effects of chronic dietary NDGA treatment on hypertriglyceridemia and hepatic steatosis, and profiled by microarray analysis the expression of enzymes and regulatory proteins involved in lipid metabolism of three insulin-sensitive tissues liver, skeletal muscle and adipose tissue. For these studies, the groups of rats were maintained on a control chow diet, HFrD or HFrD supplemented with 2.5 g NDGA/kg diet (HFrD-NDGA) (~94 mg/kg BW/24 h) for 16 weeks and, subsequently, liver, mixed gastrocnemius muscle and white adipose tissue samples were subjected to Gene Microarray Analysis. Serum samples collected at various time points were quantified for triglyceride, cholesterol, glucose and insulin levels. Chronic feeding of HFrD to male rats leads to hypertension, hyperinsulinemia, hypertriglyceridemia and inflammation [47, 56, 68].

**Fig. 1:**
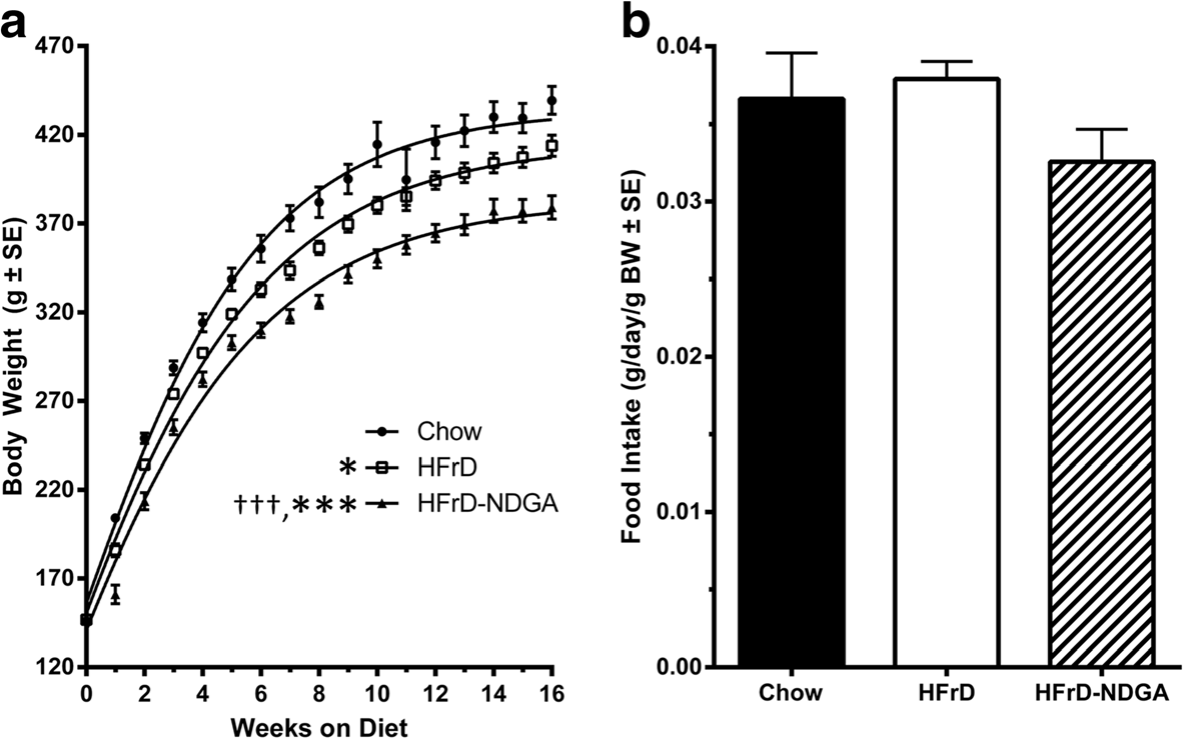
Effects of dietary administration of NDGA on body weight and diet consumption. **a** Body weight and **b** diet consumption in rats fed a chow diet (*n* = 8), HFrD (*n* = 12) or HFrD-NDGA (2.5 g/kg diet, *n* = 14)) for 8 weeks. Results are means ± SE. * *p* < 0.05, ** *p* < 0.01, *** *p* < 0.001 vs Chow, † *p* < 0.05, †† *p* < 0.01, ††† *p* < 0.001 vs HFrD

**Fig. 2:**
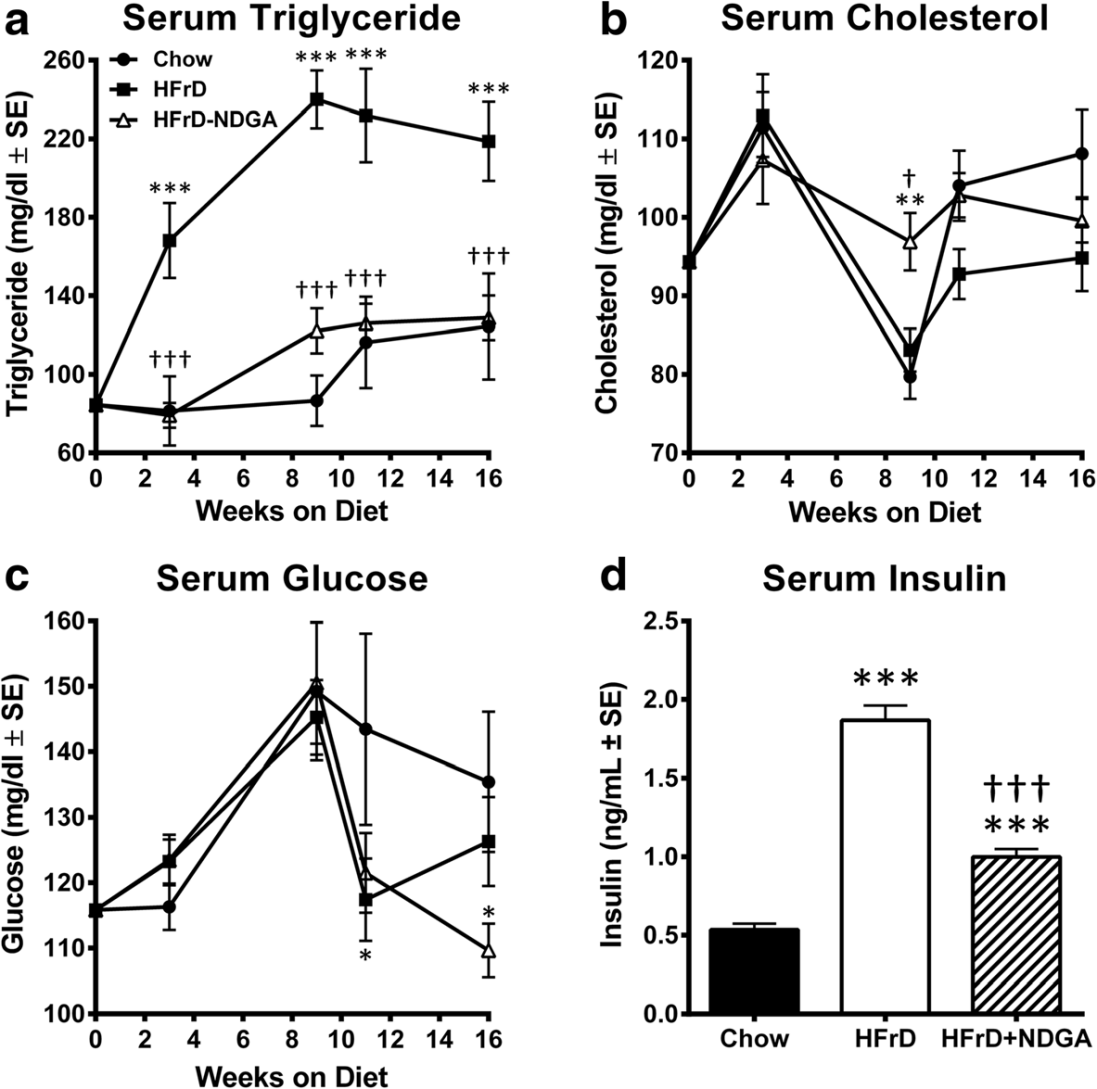
Effects of dietary administration of NDGA on plasma metabolites. **a** triglyceride, **b** total cholesterol, **c** glucose, and **d** insulin. Rats were fed a chow diet, HFrD or HFrD-NDGA (2.5 g/kg diet) for 8 weeks. Results are means ± SE. * *p* < 0.05, ** *p* < 0.01, *** *p* < 0.001 vs Chow, † *p* < 0.05, †† *p* < 0.01, ††† *p* < 0.001 vs HFrD

There was no obvious gastrointestinal stress as manifest by diarrhea observed during the dietary treatment. Compared to the chow-fed control group, there was a significant reduction of body weight starting after 1 week in both HFrD and HFrD-NDGA groups (Fig. 1[a]). Furthermore, dietary consumption of NDGA resulted in much lower body weights as compared to either the HFrD or chow diet. There was no significant difference in food intake between the groups (Fig. 1[b]). These results suggest that suppression of body weight gain in HFrD-NDGA supplementation in the diet is not due to changes in food intake, but most likely due to increased energy expenditure and decreased adiposity. HFrD-induced increases in serum triglyceride levels were completely prevented in rats maintained on HFrD supplemented with NDGA (Fig. 2[a]). However, total cholesterol (Fig. 2[b]) and glucose were not different among the three groups except at weeks 9, 11 and 16. At week 9, total cholesterol levels were significantly higher following NDGA treatment (*P* < 0.01 Chow vs HFrD-NDGA; *P* < 0.05 HFrD vs HFrD-NDGA), whereas at 11 and 16 weeks, glucose levels were reduced in response to feeding HFrD (*P* < 0.05 Chow vs HFrD) and HFrD-NDGA (*P* < 0.05 Chow vs HFrD-NDGA), respectively (Fig. 2[b, c]). As shown in Fig. 2[d], circulating insulin levels were markedly increased with HFrD feeding, but decreased to almost control levels, although were significantly different than chow with the dietary administration of NDGA (*P* < 0.001).

### Effect of chronic dietary NDGA treatment on hepatic lipid accumulation

**Fig. 3:**
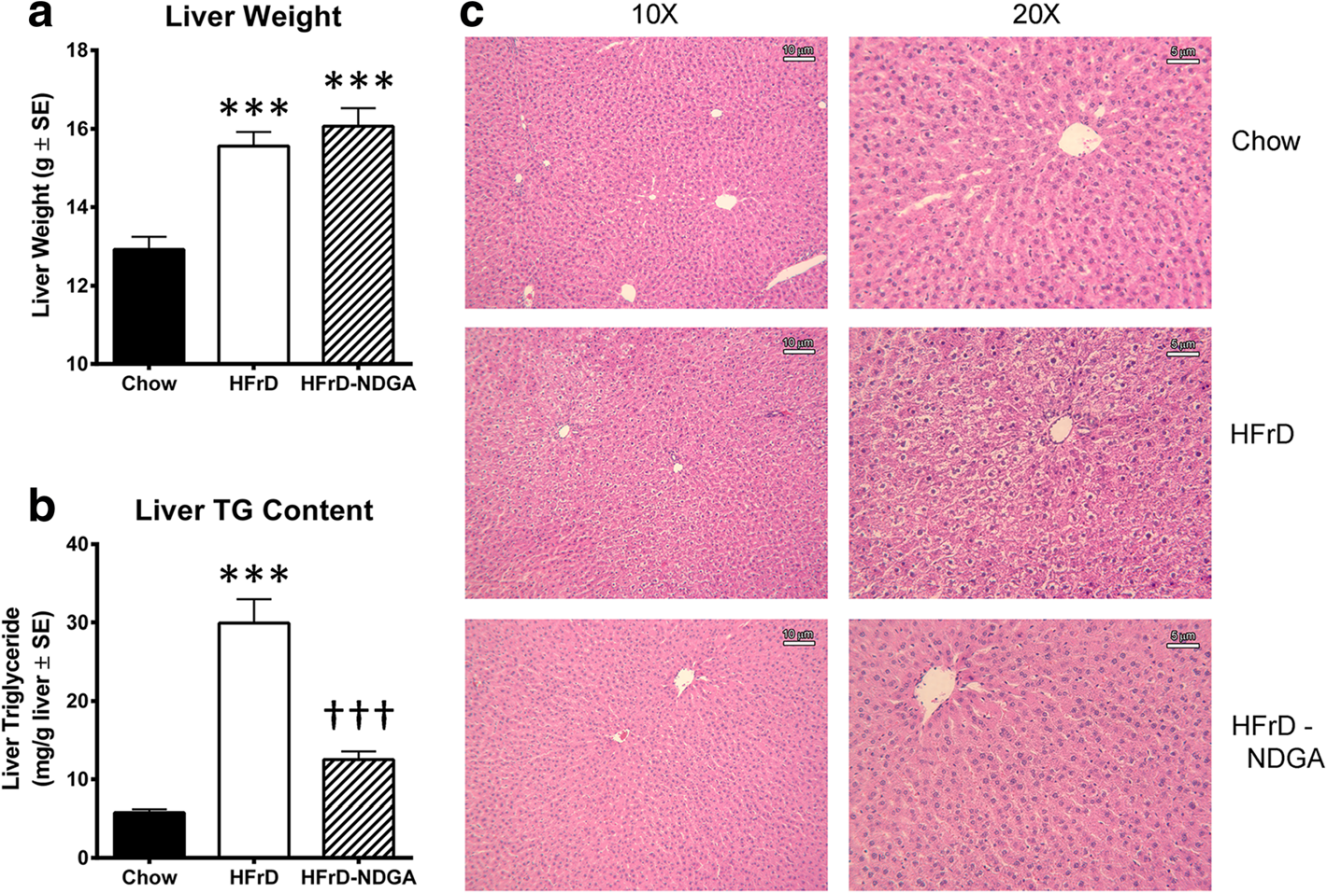
Effects of dietary administration of NDGA on liver weight, liver TG content and liver histology. **a** liver weight, **b** liver TG content, and **c** liver histology. Rats were fed a chow diet, HFrD or HFrD-NDGA (2.5 g/kg diet) for 8 weeks. Results are means ± SE. * *p* < 0.05, ** *p* < 0.01, *** *p* < 0.001 vs Chow, † *p* < 0.05, †† *p* < 0.01, ††† *p* < 0.001 vs HFrD

**Fig. 4:**
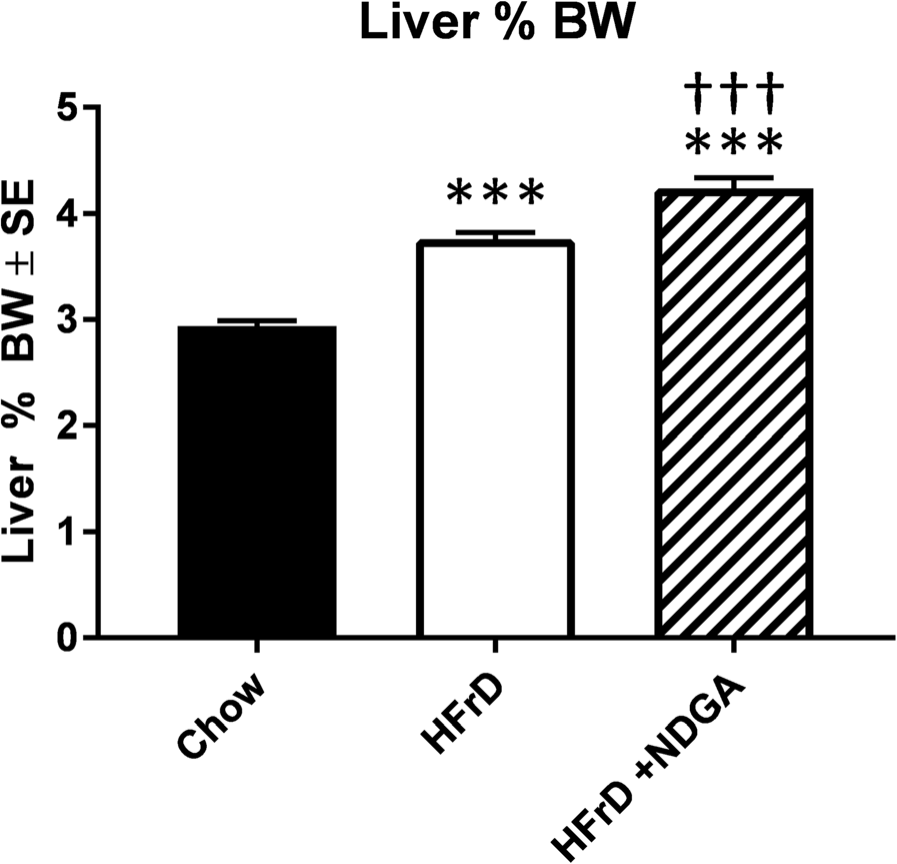
Liver/body weight ratios for rats fed a Chow diet, HFrD or HFrD-NDGA. Liver/body weight ratios were calculated from the data presented in Figs. 1 and 3. Results are means ± SE. * *p* < 0.05, ** *p* < 0.01, ***p < 0.001 vs Chow, † *p* < 0.05, †† *p* < 0.01, ††† *p* < 0.001 vs HFrD

Liver weights were significantly higher in both HFrD and HFrD-NDGA groups than controls, but no differences were noted between HFrD and HFrD-NDGA groups (Fig. 3[a]). Liver TG content increased approximately 6-fold in animals fed HFrD compared with control rats (*P* <0.001) and decreased by approximately 70 % in response to HFrD-NDGA feeding (*P* <0.001) (Fig. 3[b]). We also performed histological examinations on H&E stained liver sections prepared from chow, HFrD and HFrD-NDGA groups to assess the extent of hepatic steatosis. Hepatocytes from HFrD fed rats contained more lipids (white space) than control rats (Fig. 3[c]). In comparison, the extent of steatosis in liver tissue of rats fed HFrD-NDGA was reduced to the levels seen in chow-fed animals (Fig. 3[c]). We also calculated liver/body weight ratios. As can be seen in Fig. 4, HFrD + NDGA/body weight ratio was higher than HFrD/Body weight ratio. We speculate that this occurs because of increased hepatic glycogen accumulation, since livers of NDGA treated animals preferentially use fatty acids as an energy source. Similar results have been reported by Lee et al. [57] showing that high-fat diet (HFD) mice treated with NDGA had a higher liver body weight ratio than mice fed HFD alone.

### Principal component analysis of microarrays

**Fig. 5:**
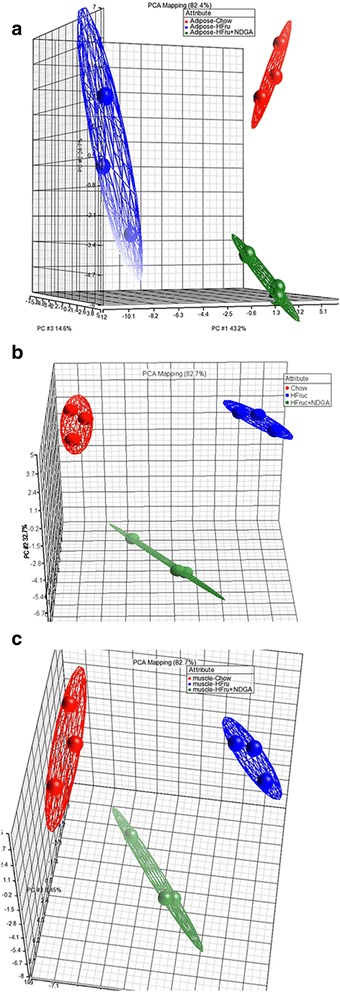
Principal component analysis (PCA) of gene expression profiles of white adipose tissue [**a**], liver [**b**] and mixed gastrocnemius muscle [**c**] samples from chow, HFrD and HFrD-NDGA fed animals

Initially a principal component analysis (PCA) was performed to visualize patterns in the gene expression data and to highlight similarities and differences between the two diets (Chow vs HFrD) and NDGA treatment (HFrD vs HFrD-NDGA). Data are displayed in a simple Scatter Plot view of the first two principal components (PCs) (Fig. 5 [a-c]). In each case, all three gene clusters are well separated from each other, suggesting they are quite different.

### Effect of NDGA on expression of genes involved in lipid metabolism in liver, skeletal muscle and adipose tissue

To examine which components of hepatic metabolism were impacted by NDGA treatment in HFrD fed rats, we categorized genes involved in lipid metabolism into eight groups: fatty acid uptake and transport, acyl-CoA synthases, fatty acid oxidation, fatty acid synthesis (lipogenesis), TG synthesis and VLDL-TG assembly, cholesterol metabolism, lipid clearance, and key transcription factors involved in the regulation of genes of lipid metabolism. As can be seen from the results presented in Table 1, NDGA treatment robustly altered the genes of hepatic fatty acid oxidation and lipogenesis. Among the fourteen fatty acid oxidation genes analyzed, expression of six genes, including *Cpt1b*, *Cpt2*, *Acox*, *Acadvl*, *Dci* and *Ehhadh*, was significantly upregulated in response to NDGA treatment of high-fructose diet fed rats. The expression of *Acadsb*, however, was down-regulated following dietary administration of NDGA. Of the sixteen lipogenic enzyme genes examined, the expression levels of *Gckr*, *Gck*, *Acly*, *Fasn*, *Scd1*, *Fads1*, *Fads2*, *Elovl2* and *Elovl5* were down-regulated, whereas expression levels of *Mlycd*, *Elovl4* and *Elovl6* were up-regulated in response to dietary consumption of both HFrD and NDGA as compared to HFrD alone. On the other hand, feeding high-fructose diet alone increased the expression of a number of lipogenic genes, including *Gckr*, *Gck*, *Pklr*, *Acyl*, *Fasn*, *Scd1*, *Fads1*, *Fads2*, *Elovl2*, *Elovl5*, and *Elovl6*.

**Table 1:** Hepatic genes responsive to feeding of a high-fructose diet (HFrD) or HFrD supplemented with NDGA (HFrD-NDGA)

Gene symbol/GenBank accession/Gene ID	Entrez gene name	HFrD/Chow (Fold-change)	HFrD-NDGA/HFrD (Fold-change)
	Fatty Acid Transport		
*Slc27a1* Acc: NM_053580.2 ID: 94172	Soluble carrier family 27 (fatty acid transporter), member 1 (FATP1)	0.817382 (*p* = 0.050249)	1.085686 (*p* = 0.548792)
*Slc27a2* Acc: NM_031736.1 ID: 65192	Soluble carrier family 27 (fatty acid transporter), member 2 (FATP2)	1.276261↑ (*p* = 0.017602)	1.071673 (*p* = 0.458057)
	Acyl-CoA Synthetases		
*Acsl1* Acc: NM_012820.1 ID: 25288	Acyl-CoA synthetase long-chain family member 1 (ACS, Acas, COAA, Facl2)	1.058003 (*p* = 0.59888)	1.286236↑ (*p* = 0.015343)
*Acsl4* Acc: NM_053623.1 ID: 113976	Acyl-CoA synthetase long-chain family member 4 (ACS4, Facl4)	1.673512↑ (*p* = 0.000004)	0.697139↓ (*p* = 0.001224)
*Acsm3* Acc: NM_033231.1 ID: 24763	Acyl-CoA synthetase medium-chain family member 3 (Sa, Sah)	0.458851↓ (*p* = 0.000004)	1.583156↑ (*p* = 0.000039)
*Acss2* Acc: NM_001107793.1 ID: 311569	Acyl-CoA synthetase short chain family member 2 (Acss2)	1.640823↑ (*p* = 5.92E-09)	0.707869↓ (*p* = 0.000022)
	Fatty Acid Oxidation		
*Cpt1a* Acc: NM_031559.2 ID: 25757	Carnitine palmitoyltransferase 1a, liver (CPT-1a)	2.093217↑ (*p* = 0.000002)	0.935127 (*p* = 0.569094)
*Cpt1b* Acc: NM_013200.1 ID: 25756	Carnitine palmitoyltransferase 1b, muscle (CPT-1B, M-CPT1)	0.955041 (*p* = 0.714963)	4.901597↑ (*p* = 1.32E-23)
*Cpt1c* Acc: NM_001034925.2 ID: 308579	Carnitine palmitoyltransferase 1c (CPT 1C, CPT1-B, CPTI-B)	0.786901 (*p* = 0.063481)	0.809912 (*p* = 0.13046)
*Cpt2* Acc: NM_012930.1 ID: 25413	Carnitine palmitoyltransferase 2 (CPTII)	1.119695 (*p* = 0.344743)	2.583082↑ (*p* = 1.88E-16)
*Acox1* Acc: NM_017340.2 ID: 506811	Acyl-CoA oxidase, palmitoyl (Rat ACOA1)	1.116564 (*p* = 0.176539)	3.27028↑ (*p* = 5.20E-32)
*Acadl* Acc: NM_012819.1 ID: 25287	Acyl-CoA dehydrogenase, long chain (ACOADA, LCAD)	1.156082 (*p* = 0.178078)	1.023617 (*p* = 0.843391)
*Acadm* Acc: NM_016986.2 ID: 24158	Acyl-CoA dehydrogenase, C-4 to C-12 straight chain (MCAD)	0.871033 (*p* = 0.206145)	1.171654 (*p* = 0.099023)
*Acads* Acc: NM_022512.1 ID: 64304	Acyl-Coa dehydrogenase, C-2 to C-3 short chain (Scad)	1.125432 (*p* = 0.554859)	0.919627 (*p* = 0.563538)
*Acadsb* Acc: NM_013084.1 ID: 25618	Acyl-CoA dehydrogenase, short/branched chain	1.060392 (*p* = 0.645381)	0.700742↓ (*p* = 0.030057)
*Acadvl* Acc: NM_012891.1 ID: 25363	Acyl-CoA dehydrogenase very long chain (VLCAD)	1.034174 (*p* = 0.763361)	1.322826↑ (*p* = 0.003236)
*Eci1* Acc: NM_017306.4 ID: 29740	Enoyl-CoA delta isomerase 1(Deci)	0.991668 (*p* = 0.91246)	2.461649↑ (*p* = 1.48E-11)
*Echs1* Acc: NM_078623.2 ID: 140547	Enoy-CoA hydratase, short chain 1, mitochondrial	1.001718 (*p* = 0.984367)	0.982888 (*p* = 0.872986)
*Ehhadh* Acc: NM_133606.1 ID: 171142	Enoyl-CoA hydratase/3-hydroxyacyl-CoA dehydrogenase	1.159127 (*p* = 0.181858)	5.837078↑ (*p* = 2.67E-36)
*Eci2* Acc: NM_001006966.1 ID:291075	Enoyl-CoA delta isomerase 2 (Peci)	1.109171 (*p* = 0.294883)	1.04819 (*p* = 0.563012)
	Fatty Acid Synthesis/ De Novo Lipogenesis		
Gckr Acc: NM_013120.2 ID: 25658	Glucokinase (hexokinase 4) regulator (GLRE)	1.225835↑ (*p* = 0.00735)	0.842312↓ (*p* = 0.029236)
*Gck* Acc: NM_012565.1 ID: 24385	Glukokinase (GLUKA, RNGK2)	1.954322↑ (*p* = 2.09E-18)	0.6409↓ (*p* = 9.91E-08)
*Pklr* Acc: NM_012624.3 ID: 24651	Pyruvate kinase, liver and RBC (PK1, PKL, Pklg)	2.101247↑ (*p* = 0.013312)	0.763246 (*p* = 0.331269)
*Acly* Acc: NM_016987.2 NM_001111095.1 ID: 24159	ATP citrate lyase (ACL, Clatp)	3.860112↑ (*p* = 8.66E-38)	0.669940↓ (*p* = 0.000006)
*Fasn* Acc: NM_01332.1 ID: 50761	Fatty acid synthase	8.556156↑ (*p* = 00E + 00)	0.448329↓ (*p* = 2.08E-16)
*Me1* Acc: NM_012600.2 ID: 24552	Malic enzyme 1, NADP(+)-dependent, cytosolic (MOD1)	8.437617↑ (*p* = 2.81E-20)	1.132866 (*p* = 0.447740)
*Me2* Acc: NM_001111095.1 ID: 307270	Malic enzyme 2, NAD(+)-dependent, mitochondrial	—	—
*Scd1* Acc: NM_139192.2 ID: 246074	Stearyol-Coenzyme A desaturase 1	9.689369↑ (*p* = 1.82E-38)	0.563616↓ (*p* = 3.64E-11)
*Elovl1* Acc: NM_001044275.1 ID: 67953.2	ELOVL fatty acid elongase 1	0.996626 (*p* = 0.968014) and 0.943940 (*p* = 0.460163)	0.956685 (*p* = 0.573719) and 1.033276 (*p* = 0.664855)
*Elovl2* Acc: NM_001109118.1 ID: 498728	ELOVL fatty acid elongase 2	2.461290↑ (*p* = 6.36E-19)	0.498430↓ (*p* = 3.66E-11)
*Elovl4* Acc: NM_001191796.1 XM_001062735.2 ID: 315851	ELOVL fatty acid elongase 4	0.823072 (*p* = 0.428065)	1.658203↑ (*p* = 0.022936)
*Elovl5* Acc: NM_134382.1 ID: 171400	ELOVL fatty acid elongase 5 (rELO1)	1.898944↑ (*p* = 1.23E-11)	0.416035↓ (*p* = 1.73E-17)
*Elovl6* Acc: NM_134383.2 ID: 171402	ELOVL fatty acid elongase 6 (Lce2, rELO2)	9.009938↑ (*p* = 0.00E + 00) and 2.808326↑ (*p* = 5.04E-11)	1.516732↑ (*p* = 6.34E-09) and 1.466229↑ (*p* = 0.000012)
*Fads1* Acc: NM_053445.2 ID: 84575	Fatty acid desaturase 1	1.241893↑ (*p* = 0.044164)	0.760081↓ (*p* = 0.006978)
*Fads2* Acc: NM_031344.2 ID: 83512	Fatty acid desaturase 2 (Fadsd6)	2.161421↑ (*p* = 8.64E-12)	0.699797↓ (*p* = 0.000734)
*Mlcyd* Acc: NM_053477.1 ID: 85239	Malonyl-CoA decarboxylase	1.018118 (*p* = 0.862908)	1.421141↑ (*p* = 0.000017)
	Triglyceride (TG) Synthesis/VLDL-TG Assembly		
*Agpat1* Acc: NM_212458.1 ID: 406165	1-Acylglycerol-3-phosphate *O*-acyltransferase 1	0.695809↓ (*p* = 0.00083)	0.906865 (*p* = 0.595394)
*Agpat2* Acc: NM_001107821.1 ID: 311821	1-Acylglycerol-3-phosphate *O*-acyltransferase 2	0.846901 (*p* = 0.109629)	1.048686 (*p* = 0.622341)
*Agpat3* Acc: NM_001106378.1 ID: 311821	1-Acylglycerol-3-phosphate *O*-acyltransferase 3	0.848841 (*p* = 0.540663)	0.895847 (*p* = 0.800248)
*Agpat6* Acc: NM_001047849.1 ID: 305166	1-Acylglycerol-3-phosphate O-acyltransferase 6 (RGD1310520)	1.024470 (*p* = 0.740343)	0.977072 (*p* = 0.773786)
Agpat9 Acc: NM_001025670.1 ID: 305166	1-Acylglycerol-3-phosphate *O*-acyltransferase 9	─	─
*Mogat1* Acc: NM_001108803.1 ID: 363261	Monoacylglycerol *O*-acyltransferase 1	0.682029 (*p* = 0.154868)	1.75896↑ (*p* = 0.030197)
*Dgat1* Acc: NM_053437.1 ID: 84497	Diacylglycerol *O*-acyltransferase 1 (ARAT, Dgat)	1.222808↑ (*p* = 0.030522)	1.067215 (*p* = 0.527579)
*Dgat2* Acc: NM_001012345.1 ID: 252900	Diacylglycerol *O*-acyltransferase 2 (ARAT)	1.171782 (*p* = 0.67572)	0.664628↓ (*p* = 0.000124)
*Arf3* Acc: NM_080904.2 ID: 140940	ADP-ribosylation factor 3 (AC1-253) Cholesterol Synthesis/Metabolism	1.046754 (*p* = 0.67572)	0.743228↓ (*p* = 0.001530)
	Cholesterol Synthesis/Metabolism		
*Acat2* Acc: NM_001006995.1 ID: 308100	Acetyl-CoA acetyltransferase 2 (Acat3, Ab2-076)	0.944516 (*p* = 0.461984)	0.957373 (*p* = 0.56688)
*Hmgcr* Acc: NM_013134.2 ID: 25675	3-Hydroxy-3-methylglutaryl-CoA reductase (3H3M)	1.438208↑ (*p* = 0.000036)	0.735862↓ (*p* = 0.002838)
*Insig 1* Acc: NM_022392.1 ID: 64194	Insulin induced gene 1	2.844270↑ (*p* = 9.18E-16)	0.542157↓ (*p* = 0.000001)
*Insig 2* Acc: NM_178091.4 ID: 288985	Insulin induced gene 2	0.831160↓ (*p* = 0.044277)	0.630641↓ (*p* = 8.36E-09)
*Ldlr* Acc: NM_175762.2 ID: 300438	Low density lipoprotein receptor (LDLRA)	1.439569↑ (*p* = 0.003984)	0.840219 (*p* = 0.137488)
*Mvk* Acc: NM_031063.1 ID: 81727	Mevalonate kinase (Lrbp)	0.663626↓ (*p* = 0.000016)	1.149403 (*p* = 0.234165)
*Scap* Acc: NM_001100966.1 ID: 301024	SREBF chaperone Proteins Involved in Lipid Clearance	1.171144↑ (*p* = 0.035192)	0.995421 (*p* = 0.941247)
	Proteins Involved in Lipid Clearance		
*Abca4* Acc: NM_00110772.1 ID: 310836	ATP-binding cassette, subfamily A (ABC1), member 4 (ABCR)	─	─
*Abcb4* Acc: NM_012690.1 ID: 24891	ATP-binding cassette, subfamily B (MDR/TAP), member 4 (Mdr2, Pgy3)	0.489385↓ (*p* = 3.69E-12)	1.099759 (*p* = 0.27820)
*Abcb11* Acc: NM_031760.1 ID: 83569	ATP-binding cassette, subfamily B (MDR/TAP), member 11 (Bsep, Spgp)	0.752953↓ (*p* = 0.001332)	1.158787 (*p* = 0.07294)
*Abcc3* Acc: NM_080581.1 ID: 140668	ATP-binding cassette, subfamily C (CFTR/MRP), member 3 (Mlp2, Mrp3)	0.813337 (*p* = 0.08045)	2.659410↑ (*p* = 7.16E-11)
*Abcc6* Acc: NM_031013.1 ID: 81642	ATP-binding cassette, subfamily C (CFTR/MRP), member 6 (Mrp6)	1.085723 (*p* = 0.31384)	0.717441↓ (*p* = 0.00001)
*Abcd1* Acc: NM_001108821.1 ID: 363516	ATP-binding cassette, subfamily D (ALD), member 3 (RGD1562128)	1.087157 (*p* = 0.468224)	0.943107 (*p* = 0.56163)
*Abcd3* Acc: NM_012804.1 ID: 25270	ATP-binging cassette, subfamily D (ALD), member 3 (PMP70, Pxmp1)	1.178867↑ (*p* = 0.035741)	2.15542↑ (*p* = 2.62E-16)
*Abcg2* Acc: NM_181381.2 ID: 312382	ATP-binding cassette, subfamily G (WHITE), member 2 (BCRP1)	1.940981↑ (*p* = 0.000000)	1.140117 (*p* = 0.258891)
*Abcg5* Acc: NM_053754.2 ID: 114628	ATP-binding cassette, subfamily G (WHITE), member 5	0.297237↓ (*p* = 5.23E-28)	3.499936↑ (*p* = 5.81E-28)
*Apoa4* Acc: NM_012737.1 ID: 25080	Apolipoprotein A-IV (Apo-AIV, ApoA-IV, ApoAIV)	1.316723↑ (*p* = 0.025675)	0.447885↓ (*p* = 1.8E-10)
*Apob* Acc: NM_019287.2 ID: 54225	Apolipoprotein B (Aa1064, AC1-060, Apo B-100, ApoB-100, ApoB-48)	0.895361 (*p* = 0.277000)	1.130493 (*p* = 0.142599)
*Apoc2* Acc: NM_001085352.1 ID: 292697	Apolipoprotein C-II (RGD1560725)	0.981675 (*p* = 0.864333)	0.941418 (*p* = 0.591544)
*Apoc3* Acc: NM_012501.1 ID: 24207	Apolipoprotein C-III (ApoC-III, apo-CIII)	0.887282 (*p* = 0.221273)	1.133806 (*p* = 0.221176)
*Apoe* Acc: NM_138828.2 ID: 25728	Apolipoprotein E (APOEA)	0.953286 (*p* = 0.543392)	0.968783 (*p* = 0.800654)
*Apof* Acc: NM_001024351.1 ID: 500761	Apolipoprotein F	1.053397 (*p* = 0.630710)	0.558207↓ (*p* = 0.000001)
*Lpl* Acc: NM_012498.2 ID: 24539	Lipoprotein lipase	1.300129↑ (*p* = 0.024577)	3.799195↑ (*p* = 1.34E-16)
*Vldlr* Acc: NM_013155.2 ID: 25696	Very low density lipoprotein receptor	0.993145 (*p* = 0.973839) Or 1.103786 (*p* = 0.800081)	1.615341↑ (*p* = 0.012976) Or 1.454645 (*p* = 0.304421)
*Ppt1* Acc: NM_022502.2 ID: 29411	Palmitoyl-protein thioesterase 1 (Ppt)	1.055246 (*p* = 0.5199114)	0.882921 (*p* = 0.192733)
	Central Metabolic Regulators (Lipid Transcription Factors)		
*Foxa1* Acc: NM_012742.1 ID: 25098	Forkhead box A1	1.489049↑ (*p* = 0.001314) Or 1.271372↑ (*p* = 037977)	0.831777 (*p* = 0.136951) Or 0.645434↓ (p = 0.000052)
*Hnf4a* Acc: NM_022180.1 ID: 25735	Hepatocyte nuclear factor 4, alpha (Hnf4alpha, Hnf4a)	0.912061 (*p* = 0.377142)	1.357585↑ (*p* = 0.003965)
Mlxipl Acc: NM_133552.1 ID: 171078	MLX interacting protein-like (ChREBP, WS-bHLH, Wbscr14, bHLHd14)	1.976840↑ (*p* = 1.9E-15)	0.724559↓ (*p* = 0.000044)
*Nr1h3* Acc: NM_031627.2 ID: 58852	Nuclear receptor subfamily 1, group H, member 3 (LXRalpha)	0.980054 (*p* = 0.831777)	1.208346↑ (*p* = 0.041273)
*Ppara* Acc: NM_013196.1 ID: 25747	Peroxisome proliferator activated receptor alpha (PPAR)	1.009554 (*p* = 0.946257)	1.760026↑ (*p* = 0.000067)
*Ppard* Acc: NM_013141.2 ID: 25682	Peroxisome proliferator activated receptor delta (Pparb)	0.843786 (*p* = 0.226207) and 1.189417 (*p* = 0.204488)	0.753122 (*p* = 0.144411) and 0.635361↓ (*p* = 0.029129
*Pparg* Acc: NM_013124.3 NM_001145366.1 NM_001145367.1 ID: 25664	Peroxisome proliferator activated receptor gamma	1.010459 (*p* = 0.879959)	1.03214 (*p* = 0.742011)
*Ppargc1a* Acc: NM_031347.1 ID: 83516	Peroxisome proliferator activated receptor gamma, coactivator 1 alpha(Ppargc1)	0.738155 (*p* = 0.120955)	─
*Ppargc1b* Acc: NM_176075.2 ID: 291567	Peroxisome proliferator activated receptor, coactivator 1 beta (PGC1beta, Perc)	0.969931 (*p* = 0.815666)	1.534911↑ (*p* = 0.012486)
*Srebf1* Acc: XM_213329.5 XM_001075680.2 ID: 78968	Sterol regulatory element binding transcription factor 1 (ADD-1, ADD1, SREBP-1, SREBP-1c, Srebp1)	1.604384↑ (*p* = 0.000000) and 1.456690↑ (*p* = 0.001744)	0.8782 (*p* = 0.125948) and 0.934259 (*p* = 0.479001)
Srebf2 Acc: NM_001033694.1 ID: 300095	Sterol regulatory element binding transcription factor 2 (SREBP-2, SREBP2-retired, Srebf2)	0.732025↓ (*p* = 0.000761)	1.071545 (*p* = 0.478687)
*Xbp1* Acc: NM_001004210.1 ID: 289754	X-box binding protein 1 (HTF)	1.148942 (*p* = 0.076252)	0.807070↓ (*p* = 0.016697)

The expression of acyl-CoA synthetases, which channel fatty acids into different pathways of lipid metabolism, was differentially impacted by NDGA treatment; NDGA treatment increased *Acsl1* and *Acsm3* mRNA levels, but decreased *Acsl4* and *Acss2* levels (Table 1). Likewise, NDGA treatment differentially increased *Abcc3*, *Abcd3*, *Abcg5*, *Lpl*, and *Vldr* and decreased *Abcc6*, *Apoa4*, *Apof*, genes that participate in lipid clearance. Surprisingly, NDGA showed no significant effect on various genes involved in TG synthesis/assembly with the exception of *Mogat1* (↑), *Dgat2* (↓) and *Arf3* (↓). Key transcription factors of hepatic lipid metabolism, such as *Hnf4a*, *Ppara*, *Nr1h3* and *Ppargc1b*, showed enhanced expression, whereas *Foxa1*, *Mlxipl*, *Srebf1*, *Ppard* and *Xbp1* expression was significantly reduced in HFrD-NDGA treated animals as compared to HFrD treated animals (Table 1).

Qualitatively similar to hepatic gene expression, data were obtained for the mixed gastrocnemius muscle, a representative of skeletal muscle types. Expression of fatty acid oxidation genes, *Cpt1c*, *Acox1*, *Acadsb*, *Dci*, and *Ehhadh*, was up-regulated, but the expression of *Cpt1a*, *Cpt1b* and *Peci* was down-regulated with NDGA treatment (Table 2). Four fatty acid synthesizing enzyme genes (*Gckr*, *Gck*, *Fads1* and *Fads2*) were upregulated by NDGA treatment, but the expression of three genes, (*Acly*, *Elovl1* and *Elovl5*) was down-regulated in response to NDGA treatment. No NDGA effect was noted on the expression levels of key lipogenic enzymes, *Fasn* and *Scd1*. The expression levels of fatty acid transport proteins *Slc27a2* and *Slc27a1* and acyl-CoA synthetases *Acss2* and *Acsl1* were up-regulated and down-regulated, respectively, as a result of NDGA treatment. The key TG synthesizing genes *Agpat1*, *Dgat1* and *Dgat2* were increased in the HFrD-NDGA treated group compared to HFrD alone, but the expression levels of other genes, *Agpat2*, *Mogat1* and *Arf3*, were reduced with NDGA treatment. The expression levels of some genes involved in lipid clearance, *Abcb4*, *Abcb11*, *Apob*, *Apoc2*, *Apoc3* and *Vldlr*, were significantly upregulated, while levels of *Abcc3*, *Apoe* and *Lpl* were downregulated in response to NDGA treatment of HFrD fed rats. Interestingly, NDGA treatment increased the expression of both transcription factors involved in fatty acid oxidation (*Ppara* and *Ppard*) and fatty acid synthesis (*Nr1h3*, *Srebf1* and *Xbp1*) (Table 2).

**Table 2:** Skeletal muscle genes responsive to feeding of a high-fructose diet (HFrD) or HFrD supplemented with NDGA (HFrD-NDGA)

Gene symbol/GenBank accession/Gene ID	Entrez gene name	HFrD/Chow (Fold-change)	HFrD-NDGA/HFrD (Fold-change)
	Fatty Acid Transport		
*Slc27a1* Acc: NM_053580.2 ID: 94172	Soluble carrier family 27 (fatty acid transporter), member 1 (FATP1)	1.163926 (*p* = 0.067083)	0.603296↓ (*p* = 0.000002)
*Slc27a2* Acc: NM_031736.1 ID: 65192	Soluble carrier family 27 (fatty acid transporter), member 2 (FATP2)	0.8357 (*p* = 0.727766)	6.826838↑ (*p* = 7.06E-28)
	Acyl-CoA Synthetases		
*Acsl1* Acc: NM_012820.1 ID: 25288	Acyl-CoA synthetase long-chain family member 1 (ACS, Acas, COAA, Facl2)	0.929637 (*p* = 0.400503)	0.818346↓ (*p* = 0.04231)
*Acsl4* Acc: NM_053623.1 ID: 113976	Acyl-CoA synthetase long-chain family member 4 (ACS4, Facl4)	1.060922 (*p* = 0.54153)	1.353734 (*p* = 0.125592)
*Acsm3* Acc: NM_033231.1 ID: 24763	Acyl-CoA synthetase medium-chain family member 3 (Sa, Sah)	1.134565 (*p* = 0.63539)	1.518789 (*p* = 0.055276)
*Acss2* Acc: NM_001107793.1 ID: 311569	Acyl-CoA synthetase short chain family member 2 (Acss2)	1.011241 (*p* = 0.898282)	1.280275↑ (*p* = 0.002825)
	Fatty Acid Oxidation		
*Cpt1a* Acc: NM_031559.2 ID: 25757	Carnitine palmitoyltransferase 1a, liver (CPT-1a)	1.067497 (*p* = 0.448414)	0.449902↓ (*p* = 5.12E-08)
*Cpt1b* Acc: NM_013200.1 ID: 25756	Carnitine palmitoyltransferase 1b, muscle (CPT-1B, M-CPT1)	0.828098↓ (*p* = 0.041576)	0.715237↓ (*p* = 0.000001)
*Cpt1c* Acc: NM_001034925.2 ID: 308579	Carnitine palmitoyltransferase 1c (CPT 1C, CPT1-B, CPTI-B)	1.086711 (*p* = 0.58947)	1.392775↑ (*p* = 0.007650)
*Cpt2* Acc: NM_012930.1 ID: 25413	Carnitine palmitoyltransferase 2 (CPTII)	1.095366 (*p* = 0.319692)	0.935788 (*p* = 0.459054)
*Acox1* Acc: NM_017340.2 ID: 506811	Acyl-CoA oxidase, palmitoyl (RATACOA1)	0.942485 (*p* = 0.405327)	1.239981↑ (*p* = 0.026649)
*Acadl* Acc: NM_012819.1 ID: 25287	Acyl-CoA dehydrogenase, long chain (ACOADA, LCAD)	1.038506 (*p* = 0.607105)	0.897447 (*p* = 0.286828)
*Acadm* Acc: NM_016986.2 ID: 24158	Acyl-CoA dehydrogenase, C-4 to C-12 straight chain (MCAD)	1.018954 (*p* = 0.786051)	1.120494 (*p* = 0.185916)
*Acads* Acc: NM_022512.1 ID: 64304	Acyl-Coa dehydrogenase, C-2 to C-3 short chain (Scad)	0.947987 (*p* = 0.686716)	1.163831 (*p* = 0.106741)
*Acadsb* Acc: NM_013084.1 ID: 25618	Acyl-CoA dehydrogenase, short/branched chain	0.767644 (*p* = 0.056663)	1.578611↑ (*p* = 0.000470)
*Acadvl* Acc: NM_012891.1 ID: 25363	Acyl-CoA dehydrogenase very long chain (VLCAD)	1.023041 (*p* = 0.81081)	1.104065 (*p* = 0.242381)
*Eci1* Acc: NM_017306.4 ID: 29740	Enoyl-CoA delta isomerase 1(Deci)	1.028304 (*p* = 0.734099)	1.203436↑ (*p* = 0.11201)
*Echs1* Acc: NM_078623.2 ID: 140547	Enoy-CoA hydratase, short chain 1, mitochondrial	0.890438 (*p* = 0.269622)	0.970797 (*p* = 0.801854)
*Ehhadh* Acc: NM_133606.1 ID: 171142	Enoyl-CoA hydratase/3-hydroxyacyl-CoA dehydrogenase	1.255433↑ (*p* = 0.004941)	4.521567↑ (*p* = 1.81E-20)
*Eci2* Acc: NM_001006966.1 ID:291075	Enoyl-CoA delta isomerase 2 (Peci)	0.899414 (*p* = 0.120824)	0.74287↓ (*p* = 0.000344)
	Fatty Acid Synthesis/ De Novo Lipogenesis		
Gckr Acc: NM_013120.2 ID: 25658	Glucokinase (hexokinase 4) regulator (GLRE)	1.07709 (*p* = 0.694603)	2.031414↑ (*p* = 0.00004)
*Gck* Acc: NM_012565.1 ID: 24385	Glukokinase (GLUKA, RNGK2)	0.959106 (*p* = 0.801909)	1.706245↑ (*p* = 0.000131)
*Pklr* Acc: NM_012624.3 ID: 24651	Pyruvate kinase, liver and RBC (PK1, PKL, Pklg)	1.013481 (*p* = 0.932088)	1.252908 (*p* = 0.073772)
*Acly* Acc: NM_016987.2 NM_001111095.1 ID: 24159	ATP citrate lyase (ACL, Clatp)	0.998949 (*p* = 0.98697)	0.737265↓ (*p* = 0.000026)
*Fasn* Acc: NM_01332.1 ID: 50761	Fatty acid synthase	0.706952↓ (*p* = 0.000244)	0.930935 (*p* = 0.545657)
*Me1* Acc: NM_012600.2 ID: 24552	Malic enzyme 1, NADP(+)-dependent, cytosolic (MOD1)	0.822072 (*p* = 0.082114)	1.1122701 (*p* = 0.365453)
*Me2* Acc: NM_001111095.1 ID: 307270	Malic enzyme 2, NAD(+)-dependent, mitochondrial	—	—
*Scd1* Acc: NM_139192.2 ID: 246074	Stearyol-Coenzyme A desaturase 1	1.31285↑ (*p* = 0.002357)	0.761013 (*p* = 0.065913)
*Elovl1* Acc: NM_001044275.1 ID: 67953.2	ELOVL fatty acid elongase 1	1.011133 (*p* = 0.897404) and 0.943484 (*p* = 0.539378)	0.639064↓ (*p* = 5.49E-08) and 0.584865↓ (*p* = 0.000002)
*Elovl2* Acc: NM_001109118.1 ID: 498728	ELOVL fatty acid elongase 2	2.709103 (*p* = 0.626956)	1.216575 (*p* = 0.73873)
*Elovl4* Acc: NM_001191796.1 XM_001062735.2 ID: 315851	ELOVL fatty acid elongase 4	0.831144 (*p* = 0.17275)	0.905935 (*p* = 0.619544)
*Elovl5* Acc: NM_134382.1 ID: 171400	ELOVL fatty acid elongase 5 (rELO1)	0.732669↓ (*p* = 0.000051)	0.768796↓ (*p* = 0.002744)
*Elovl6* Acc: NM_134383.2 ID: 171402	ELOVL fatty acid elongase 6 (Lce2, rELO2)	1.034949 (*p* = 0.742391) and 0.974533 (*p* = 0.82477)	0.993401 (*p* = 0.969926) and 1.246617 (*p* = 0.381630)
*Fads1* Acc: NM_053445.2 ID: 84575	Fatty acid desaturase 1	1.241893↑ (*p* = 0.044164)	0.760080↓ (*p* = 0.006978)
*Fads2* Acc: NM_031344.2 ID: 83512	Fatty acid desaturase 2 (Fadsd6)	2.161421↑ (*p* = 8.64E-12)	0.699797↓ (*p* = 0.000734)
*Mlcyd* Acc: NM_053477.1 ID: 85239	Malonyl-CoA decarboxylase	1.050798 (*p* = 0.506797)	0.988309 (*p* = 0.909295)
	Triglyceride (TG) Synthesis/VLDL-TG Assembly		
*Agpat1* Acc: NM_212458.1 ID: 406165	1-Acylglycerol-3-phosphate *O*-acyltransferase 1	0.992486 (*p* = 0.954388)	1.306654↑ (*p* = 0.038298)
*Agpat2* Acc: NM_001107821.1 ID: 311821	1-Acylglycerol-3-phosphate *O*-acyltransferase 2	1.090764 (*p* = 0.284579)	0.732075↓ (*p* = 0.001118)
*Agpat3* Acc: NM_001106378.1 ID: 311821	1-Acylglycerol-3-phosphate *O*-acyltransferase 3	0.943450 (*p* = 0.5559170)	0.954861 (*p* = 0.819498)
*Agpat6* Acc: NM_001047849.1 ID: 305166	1-Acylglycerol-3-phosphate *O*-acyltransferase 6 (RGD1310520)	0.976834 (*p* = 0.754188)	1.114009 (*p* = 0.256854)
*Agpat9* Acc: 001025670.1 ID: 305166	1-Acylglycerol-3-phosphate *O*-acyltransferase 9	0.753988↓ (*p* = 0.002922)	0.791921 (*p* = 0.185478)
*Mogat1* Acc: NM_001108803.1 ID: 363261	Monoacylglycerol *O*-acyltransferase 1	0.875509 (*p* = 0.391965)	0.592216↓ (*p* = 0.000892)
*Dgat1* Acc: NM_053437.1 ID: 84497	Diacylglycerol *O*-acyltransferase 1 (ARAT, Dgat)	0.928218 (*p* = 0.359588)	1.485906↑ (*p* = 0.000110)
*Dgat2* Acc: NM_001012345.1 ID: 252900	Diacylglycerol *O*-acyltransferase 2 (ARAT)	0.911253 (*p* = 0.262778)	1.451837↑ (*p* = 0.000067)
*Arf3* Acc: NM_080904.2 ID: 140940	ADP-ribosylation factor 3 (AC1-253)	1.119282 (*p* = 0.211709)	0.530910↓ (*p* = 8.33E-12)
	Cholesterol Synthesis/Metabolism		
*Acat2* Acc: NM_001006995.1 ID: 308100	Acetyl-CoA acetyltransferase 2 (Acat3, Ab2-076)	0.952536 (*p* = 0.440569)	0.989649 (*p* = 0.940796)
*Hmgcr* Acc: NM_013134.2 ID: 25675	3-Hydroxy-3-methylglutaryl-CoA reductase (3H3M)	1.070248 (*p* = 0.412922)	0.663322↓ (*p* = 0.008738)
*Insig 1* Acc: NM_022392.1 ID: 64194	Insulin induced gene 1	1.242984↑ (*p* = 0.031151)	1.549161↑ (*p* = 0.003666)
*Insig 2* Acc: NM_178091.4 ID: 288985	Insulin induced gene 2	0.864261 (*p* = 0.073905)	1.265492 (*p* = 0.070329)
*Ldlr* Acc: NM_175762.2 ID: 300438	Low density lipoprotein receptor (LDLRA)	0.994617 (*p* = 0.974217)	0.996123 (*p* = 0.972744)
*Mvk* Acc: NM_031063.1 ID: 81727	Mevalonate kinase (Lrbp)	1.014383 (*p* = 0.892250)	1.129775 (*p* = 0.245258)
*Npc1* Acc: NM_001002025.11 ID: 266732	Niemann-Pick disease, type C1 (Cdig2)	1.002647 (*p* = 998400)	0.806589 (*p* = 0.314930)
	Proteins Involved in Lipid Clearance		
*Abca4* Acc: NM_00110772.1 ID: 310836	ATP-binding cassette, subfamily A (ABC1), member 4 (ABCR)	─	─
*Abcb4* Acc: NM_012690.1 ID: 24891	ATP-binding cassette, subfamily B (MDR/TAP), member 4 (Mdr2, Pgy3)	1.001665 (*p* = 0.990316)	1.352659↑ (*p* = 0.002415)
*Abcb11* Acc: NM_031760.1 ID: 83569	ATP-binding cassette, subfamily B (MDR/TAP), member 11 (Bsep, Spgp)	0.976717 (*p* = 0.887315)	2.314507↑ (*p* = 0.000044)
*Abcc3* Acc: NM_080581.1 ID: 140668	ATP-binding cassette, subfamily C (CFTR/MRP), member 3 (Mlp2, Mrp3)	1.13094 (*p* = 0.112959)	0.554283↓ (*p* = 3.47E-08)
*Abcc6* Acc: NM_031013.1 ID: 81642	ATP-binding cassette, subfamily C (CFTR/MRP), member 6 (Mrp6)	1.016698 (*p* = 0.931493)	2.173900↑ (*p* = 0.000012)
*Abcd1* Acc: NM_001108821.1 ID: 363516	ATP-binding cassette, subfamily D (ALD), member 3 (RGD1562128)	0.966064 (*p* = 0.685132)	1.009964 (*p* = 0.42331)
*Abcd3* Acc: NM_012804.1 ID: 25270	ATP-binging cassette, subfamily D (ALD), member 3 (PMP70, Pxmp1)	1.071230 (*p* = 0.315252)	1.178834 (*p* = 0.074443)
*Abcg2* Acc: NM_181381.2 ID: 312382	ATP-binding cassette, subfamily G (WHITE), member 2 (BCRP1)	0.940064 (*p* = 0.737683)	─
*Abcg5* Acc: NM_053754.2 ID: 114628	ATP-binding cassette, subfamily G (WHITE), member 5	0.980266 (*p* = 0.916333)	0.810473 (*p* = 506217)
*Apoa4* Acc: NM_012737.1 ID: 25080	Apolipoprotein A-IV (Apo-AIV, ApoA-IV, ApoAIV)	0.886124 (*p* = 637386)	1.397129 (*p* = 0.121159)
*Apob* Acc: NM_019287.2 ID: 54225	Apolipoprotein B (Aa1064, AC1-060, Apo B-100, ApoB-100, ApoB-48)	0.726099 (*p* = 0.275237)	5.457586↑ (*p* = 5.48E-13)
*Apoc2* Acc: NM_001085352.1 ID: 292697	Apolipoprotein C-II (RGD1560725)	0.935620 (*p* = 0.636398)	3.570020↑ (*p* = 2.22E-17)
*Apoc3* Acc: NM_012501.1 ID: 24207	Apolipoprotein C-III (ApoC-III, apo-CIII)	0.912894 (*p* = 0.302897)	8.022169↑ (*p* = 6.3E-25)
*Apoe* Acc: NM_138828.2 ID: 25728	Apolipoprotein E (APOEA)	1.177138 (*p* = 0.066475)	0.419997↓ (*p* = 1.01E-13)
*Apof* Acc: NM_001024351.1 ID: 500761	Apolipoprotein F	─	─
*Lpl* Acc: NM_012498.2 ID: 24539	Lipoprotein lipase	0.985218 (*p* = 0.852647)	0.543580↓ (*p* = 4.93E-12)
*Vldlr* Acc: NM_013155.2 ID: 25696	Very low density lipoprotein receptor	1.062438 (*p* = 0.461893) Or 0.888362 (*p* = 0.228697)	1.091786 (*p* = 0.205328) Or 1.200139↑ (*p* = 0.030468)
*Ppt1* Acc: NM_022502.2 ID: 29411	Palmitoyl-protein thioesterase 1 (Ppt)	0.959614 (*p* = 0.555592)	0.505612↓ (*p* = 5.61E-15) (*p* = 0.192733)
	Central Metabolic Regulators (Lipid Transcription Factors)		
*Foxa1* Acc: NM_012742.1 ID: 25098	Forkhead box A1	0.861648 (*p* = 0.59303)	0.790538 (*p* = 0.286379)
*Hnf4a* Acc: NM_022180.1 ID: 25735	Hepatocyte nuclear factor 4, alpha (Hnf4alpha, Hnf4a)	0.969862 (*p* = 0.899345)	0.897382 (*p* = 0.784789)
Mlxipl Acc: NM_133552.1 ID: 171078	MLX interacting protein-like (ChREBP, WS-bHLH, Wbscr14, bHLHd14)	0.644817↓ (*p* = 1.16E-08)	0.923704 (*p* = 0.439391)
*Nr1h3* Acc: NM_031627.2 ID: 58852	Nuclear receptor subfamily 1, group H, member 3 (LXRalpha)	1.002888 (*p* = 0.964985)	1.708129↑ (*p* = 0.000001)
*Ppara* Acc: NM_013196.1 ID: 25747	Peroxisome proliferator activated receptor alpha (PPAR)	0.897809 (*p* = 0.394852)	2.537218↑ (*p* = 0.000000)
*Ppard* Acc: NM_013141.2 ID: 25682	Peroxisome proliferator activated receptor delta (Pparb)	1.000165 (*p* = 0.998956) and 0.991657 (*p* = 0.964720)	1.428902↑ (*p* = 0.005855) and 1.167184 (*p* = 0.271325)
*Pparg* Acc: NM_013124.3 NM_001145366.1 NM_001145367.1 ID: 25664	Peroxisome proliferator activated receptor gamma	0.801471↓ (*p* = 0.003078)	0.924624 (*p* = 0.447736)
*Ppargc1a* Acc: NM_031347.1 ID: 83516	Peroxisome proliferator activated receptor gamma, coactivator 1 alpha (Ppargc1)	0.719326 (*p* = 0.192474)	1.479666 (*p* = 0.067544)
*Ppargc1b* Acc: NM_176075.2 ID: 291567	Peroxisome proliferator activated receptor, coactivator 1 beta (PGC1beta, Perc)	1.020089 (*p* = 0.82730)	0.633941↓ (*p* = 0.001608)
*Srebf1* Acc: XM_213329.5 XM_001075680.2 ID: 78968	Sterol regulatory element binding transcription factor 1 (ADD-1, ADD1, SREBP-1, SREBP-1c, Srebp1)	0.986205 (*p* = 0.859100) and 1.018349 (*p* = 0.853102)	1.837534↑ (*p* = 9.10E-08) and 1.865578↑ (*p* = 0.000003)
Srebf2 Acc: NM_001033694.1 ID: 300095	Sterol regulatory element binding transcription factor 2 (SREBP-2, SREBP2-retired, Srebf2)	1.028776 (*p* = 0.707159)	0.82597 (*p* = 0.471771)
*Xbp1* Acc: NM_001004210.1 ID: 289754	X-box binding protein 1 (HTF)	1.055655 (*p* = 0.434625)	1.317409↑ (*p* = 0.000651)

Compared to liver and skeletal muscle, relatively less effect of NDGA was detected on lipid metabolizing genes in white adipose tissue (Table 3). The adipose expression of fatty acid transporter *Slc27a1* was significantly reduced by high-fructose feeding, whereas its levels were markedly increased in HFrD rats treated with NDGA. Likewise, consumption of the high-fructose diet led to down-regulation of the majority of fatty acid oxidation genes except *Acox1*, whereas NDGA treatment increased expression of only *Acadsb* and *Acadvl* (Table 3). In addition, HFrD feeding increased the expression of lipogenic genes such as *Gck*, *Pklr*, *Acly*, and *Scd1*, but reduced the levels of *Elovl1*. Simultaneous HFrD and NDGA feeding decreased the expression of several lipogenic genes, e.g., *Gck*, *Pklr*, *Scd1*, and *Fads1*, but increased the expression *Elovl1*, *Elovl5* and *Elovl6*. The expression of a number of genes involved in TG synthesis, such as *Agpat1*, *Agpat3*, *Agpat6*, *Agpat9*, *Dgat1* and *Arf3*, were reciprocally regulated by HFrD feeding and HFrD-NDGA treatment. Both high-fructose feeding and combined high-fructose diet and NDGA treatment markedly altered the expression of key genes involved in lipid clearance, e.g., *Apo4*, *Apoc2*, *Apoc3*, *Apoe*, *VLDLR* and *Lpl* (Table 3). Among the lipid transcription factors, the expression of *Ppara*, *Srebf1*, and *Nr1h3* was increased following NDGA treatment, whereas high-fructose feeding alone decreased the expression of *Ppargc1b* and *Srebf1* genes. In contrast, HFrD feeding increased the expression of *Pparg1a*.

**Table 3:** Adipose tissue genes responsive to feeding of a high-fructose diet (HFrD) or HFrD supplemented with NDGA (HFrD-NDGA)

Gene symbol/GenBank accession/Gene ID	Entrez gene name	HFrD/Chow (Fold-change)	HFrD-NDGA/HFrD (Fold-change)
	Fatty Acid Transport		
*Slc27a1* Acc: NM_053580.2 ID: 94172	Soluble carrier family 27 (fatty acid transporter), member 1 (FATP1)	0.626371↓ (*p* = 0.000005)	1.243585↑ (*p* = 0.035818)
*Slc27a2* Acc: NM_031736.1 ID: 65192	Soluble carrier family 27 (fatty acid transporter), member 2 (FATP2)	─	0.843521 (*p* = 0.704995)
	Acyl-CoA Synthetases		
*Acsl1* Acc: NM_012820.1 ID: 25288	Acyl-CoA synthetase long-chain family member 1 (ACS, Acas, COAA, Facl2)	0.871766 (*p* = 0.143945)	1.022277 (p = 0.780026)
*Acsl4* Acc: NM_053623.1 ID: 113976	Acyl-CoA synthetase long-chain family member 4 (ACS4, Facl4)	0.878305 (*p* = 0.54153)	0.964436 (*p* = 0.62090)
*Acsm3* Acc: NM_033231.1 ID: 24763	Acyl-CoA synthetase medium-chain family member 3 (Sa, Sah)	0.987186 (*p* = 0.961215)	0.805024 (*p* = 0.413656)
*Acss2* Acc: NM_001107793.1 ID: 311569	Acyl-CoA synthetase short chain family member 2 (Acss2)	0.966705 (*p* = 0.717211)	0.884133 (*p* = 0.161879)
	Fatty Acid Oxidation		
*Cpt1a* Acc: NM_031559.2 ID: 25757	Carnitine palmitoyltransferase 1a, liver (CPT-1a)	0.929886 (*p* = 0.346192)	0.971389 (*p* = 0.737175)
*Cpt1b* Acc: NM_013200.1 ID: 25756	Carnitine palmitoyltransferase 1b, muscle (CPT-1B, M-CPT1)	0.705154↓ (*p* = 0.000182)	1.080327 (*p* = 0.411449)
*Cpt1c* Acc: NM_001034925.2 ID: 308579	Carnitine palmitoyltransferase 1c (CPT 1C, CPT1-B, CPTI-B)	0.912484 (*p* = 0.292825)	1.076052 (*p* = 0.61451)
*Cpt2* Acc: NM_012930.1 ID: 25413	Carnitine palmitoyltransferase 2 (CPTII)	1.058655 (*p* = 0.394189)	1.03461 (*p* = 0.630057)
*Acox1* Acc: NM_017340.2 ID: 506811	Acyl-CoA oxidase, palmitoyl (RATACOA1)	1.291301↑ (*p* = 0.000954)	1.086216 (*p* = 0.293942)
*Acadl* Acc: NM_012819.1 ID: 25287	Acyl-CoA dehydrogenase, long chain (ACOADA, LCAD)	1.024278 (*p* = 0.778232)	0.976708 (*p* = 0.800795)
*Acadm* Acc: NM_016986.2 ID: 24158	Acyl-CoA dehydrogenase, C-4 to C-12 straight chain (MCAD)	1.017514 (*p* = 0.84005)	0.917314 (*p* = 0.340538)
*Acads* Acc: NM_022512.1 ID: 64304	Acyl-Coa dehydrogenase, C-2 to C-3 short chain (Scad)	0.645793↓ (*p* = 0.000149)	1.280965 (*p* = 0.065028)
*Acadsb* Acc: NM_013084.1 ID: 25618	Acyl-CoA dehydrogenase, short/branched chain	0.775064 (*p* = 0.065038)	1.804414↑ (*p* = 0.00009)
*Acadvl* Acc: NM_012891.1 ID: 25363	Acyl-CoA dehydrogenase very long chain (VLCAD)	0.785093↓ (*p* = 0.006311)	1.285064↑ (*p* = 0.003725)
*Eci1* Acc: NM_017306.4 ID: 29740	Enoyl-CoA delta isomerase 1(Deci)	0.978982 (*p* = 0.785245)	1.134394 (*p* = 0.11201)
*Echs1* Acc: NM_078623.2 ID: 140547	Enoy-CoA hydratase, short chain 1, mitochondrial	0.764837↓ (*p* = 0.007566)	1.220593 (*p* = 0.080591)
*Ehhadh* Acc: NM_133606.1 ID: 171142	Enoyl-CoA hydratase/3-hydroxyacyl-CoA dehydrogenase	0.64736↓ (*p* = 0.000141)	1.219944 (*p* = 0.061876)
*Eci2* Acc: NM_001006966.1 ID:291075	Enoyl-CoA delta isomerase 2 (Peci)	0.899414 (*p* = 0.120824)	0.74287↓ (*p* = 0.000344)
	Fatty Acid Synthesis/ De Novo		
	Lipogenesis		
Gckr Acc: NM_013120.2 ID: 25658	Glucokinase (hexokinase 4) regulator (GLRE)	0.755501 (*p* = 0.055196)	1.126918 (*p* = 0.490323)
*Gck* Acc: NM_012565.1 ID: 24385	Glukokinase (GLUKA, RNGK2)	4.265985↑ (*p* = 6.89E-18)	0.302628↓ (*p* = 4.47E-11)
*Pklr* Acc: NM_012624.3 ID: 24651	Pyruvate kinase, liver and RBC (PK1, PKL, Pklg)	2.097495↑ (*p* = 0.000451)	0.498737↓ (*p* = 0.000675)
*Acly* Acc: NM_016987.2 NM_001111095.1 ID: 24159	ATP citrate lyase (ACL, Clatp)	1.295435↑ (*p* = 0.000403)	0.895746 (*p* = 0.143597)
*Fasn* Acc: NM_01332.1 ID: 50761	Fatty acid synthase	1.08142 (*p* = 0.423101)	0.904824 (*p* = 0.357733)
*Me1* Acc: NM_012600.2 ID: 24552	Malic enzyme 1, NADP(+)-dependent, cytosolic (MOD1)	0.847973 (*p* = 0.065373)	0.990152 (*p* = 0.920281)
*Me2* Acc: NM_001111095.1 ID: 307270	Malic enzyme 2, NAD(+)-dependent, mitochondrial	—	—
*Scd1* Acc: NM_139192.2 ID: 246074	Stearyol-Coenzyme A desaturase 1	9.92997↑ (*p* = 0.00E + 00)	0.367553↓ (p = 3.10E-28)
*Elovl1* Acc: NM_001044275.1 ID: 67953.2	ELOVL fatty acid elongase 1	0.696973↓ (*p* = 0.) and 1.025883 (*p* = 0.77018)	1.502326↑ (*p* = 5.63E-09) and 1.199776 (*p* = 0.056696)
*Elovl2* Acc: NM_001109118.1 ID: 498728	ELOVL fatty acid elongase 2	0.712072 (*p* = 0.389679)	0.330644 (*p* = 0.176175)
*Elovl4* Acc: NM_001191796.1 XM_001062735.2 ID: 315851	ELOVL fatty acid elongase 4	1.053677 (*p* = 0.719414)	0.950803 (*p* = 0.678269)
*Elovl5* Acc: NM_134382.1 ID: 171400	ELOVL fatty acid elongase 5 (rELO1)	1.027579 (*p* = 0.719414)	2.101966↑ (*p* = 1.14E-17)
*Elovl6* Acc: NM_134383.2 ID: 171402	ELOVL fatty acid elongase 6 (Lce2, rELO2)	0.511033↓ (*p* = 2.11E-11) and 1.322709↑ (*p* = 0.037357)	1.436011↑ (*p* = 0.001273) and 0.797587 (*p* = 0.0795)
*Fads1* Acc: NM_053445.2 ID: 84575	Fatty acid desaturase 1	1.16415 (*p* = 0.102452)	0.828712↓ (*p* = 0.042428)
*Fads2* Acc: NM_031344.2 ID: 83512	Fatty acid desaturase 2 (Fadsd6)	1.036468 (*p* = 0.84278)	0.890867 (*p* = 0.485008)
*Mlcyd* Acc: NM_053477.1 ID: 85239	Malonyl-CoA decarboxylase	1.06015 (*p* = 0.581558)	0.800024↓ (*p* = 0.047506)
	Triglyceride (TG) Synthesis/VLDL-TG Assembly		
*Agpat1* Acc: NM_212458.1 ID: 406165	1-Acylglycerol-3-phosphate *O*-acyltransferase 1	1.733174↑ (*p* = 0.000011)	0.551598↓ (*p* = 0.000015)
*Agpat2* Acc: NM_001107821.1 ID: 311821	1-Acylglycerol-3-phosphate *O*-acyltransferase 2	0.890825 (*p* = 0.154834)	0.98959 (*p* = 0.895733)
*Agpat3* Acc: NM_001106378.1 ID: 311821	1-Acylglycerol-3-phosphate *O*-acyltransferase 3	0.625706↓ (*p* = 0.004796)	1.138055 (*p* = 0.352178)
*Agpat6* Acc: NM_001047849.1 ID: 305166	1-Acylglycerol-3-phosphate *O*-acyltransferase 6 (RGD1310520)	0.668305↓ (*p* = 0.754188)	1.483745↑ (*p* = 0.000001)
*Agpat9* Acc: 001025670.1 ID: 305166	1-Acylglycerol-3-phosphate *O*-acyltransferase 9	0.702126↓ (*p* = 0.007365)	1.461539↑ (*p* = 0.002906)
*Mogat1* Acc: NM_001108803.1 ID: 363261	Monoacylglycerol *O*-acyltransferase 1	0.911577 (*p* = 0.405954)	0.977825 (*p* = 0.860977)
*Dgat1* Acc: NM_053437.1 ID: 84497	Diacylglycerol *O*-acyltransferase 1 (ARAT, Dgat)	0.766526↓ (*p* = 0.033377)	1.35964↑ (*p* = 0.012554)
*Dgat2* Acc: NM_001012345.1 ID: 252900	Diacylglycerol *O*-acyltransferase 2 (ARAT)	1.000764 (*p* = 0.992838)	0.890682 (*p* = 0.159626)
*Arf3* Acc: NM_080904.2 ID: 140940	ADP-ribosylation factor 3 (AC1-253)	1.253088↑ (*p* = 0.000991)	0.745999↓ (*p* = 0.000289)
	Cholesterol Synthesis/Metabolism		
*Acat2* Acc: NM_001006995.1 ID: 308100	Acetyl-CoA acetyltransferase 2 (Acat3, Ab2-076)	1.149942 (*p* = 0.064026)	0.846982↓ (*p* = 0.020354)
*Hmgcr* Acc: NM_013134.2 ID: 25675	3-Hydroxy-3-methylglutaryl-CoA reductase (3H3M)	0.614003↓ (*p* = 0.000734)	1.212062 (*p* = 0.155741)
*Insig 1* Acc: NM_022392.1 ID: 64194	Insulin induced gene 1	1.046145 (*p* = 0.573908)	0.618888↓ (*p* = 0.000006)
*Insig 2* Acc: NM_178091.4 ID: 288985	Insulin induced gene 2	0.823541 (*p* = 0.133816)	1.467193↑ (*p* = 0.003866)
*Ldlr* Acc: NM_175762.2 ID: 300438	Low density lipoprotein receptor (LDLRA)	0.761982 (*p* = 0.148211)	1.204468 (*p* = 0.362466)
*Mvk* Acc: NM_031063.1 ID: 81727	Mevalonate kinase (Lrbp)	1.024571 (*p* = 0.839357)	1.168772 (*p* = 0.150024)
*Npc1* Acc: NM_001002025.11 ID: 266732	Niemann-Pick disease, type C1 (Cdig2)	0.838672 (*p* = 0.861612)	1.21535 (*p* = 0.847542)
	Proteins Involved in Lipid Clearance		
*Abca4* Acc: NM_00110772.1 ID: 310836	ATP-binding cassette, subfamily A (ABC1), member 4 (ABCR)	─	─
*Abcb4* Acc: NM_012690.1 ID: 24891	ATP-binding cassette, subfamily B (MDR/TAP), member 4 (Mdr2, Pgy3)	1.290231 (*p* = 0.284859)	0.894225 (*p* = 0.438512)
*Abcb11* Acc: NM_031760.1 ID: 83569	ATP-binding cassette, subfamily B (MDR/TAP), member 11 (Bsep, Spgp)	0.693218 (*p* = 0.05412)	1.397109 (*p* = 0.086006)
*Abcc3* Acc: NM_080581.1 ID: 140668	ATP-binding cassette, subfamily C (CFTR/MRP), member 3 (Mlp2, Mrp3)	0.930144 (*p* = 0.499966)	0.930223 (*p* = 0.509623)
*Abcc6* Acc: NM_031013.1 ID: 81642	ATP-binding cassette, subfamily C (CFTR/MRP), member 6 (Mrp6)	1.044104 (*p* = 0.931493)	0.953551 (*p* = 0.836794)
*Abcd1* Acc: NM_001108821.1 ID: 363516	ATP-binding cassette, subfamily D (ALD), member 3 (RGD1562128)	0.981591 (*p* = 0.867544)	0.973671 (*p* = 0.807543)
*Abcd3* Acc: NM_012804.1 ID: 25270	ATP-binging cassette, subfamily D (ALD), member 3 (PMP70, Pxmp1)	0.775986↓ (*p* = 0.000407)	1.067058 (*p* = 0.3708)
*Abcg2* Acc: NM_181381.2 ID: 312382	ATP-binding cassette, subfamily G (WHITE), member 2 (BCRP1)	0.840673 (*p* = 0.694657)	1.966469 (*p* = 0.051575)
*Abcg5* Acc: NM_053754.2 ID: 114628	ATP-binding cassette, subfamily G (WHITE), member 5	1.289574 (*p* = 0.31897)	0.839246 (*p* = 0.375668)
*Apoa4* Acc: NM_012737.1 ID: 25080	Apolipoprotein A-IV (Apo-AIV, ApoA-IV, ApoAIV)	1.975251↑ (*p* = 0.005407)	0.701419 (*p* = 0.119689)
*Apob* Acc: NM_019287.2 ID: 54225	Apolipoprotein B (Aa1064, AC1-060, Apo B-100, ApoB-100, ApoB-48)	0.90111 (*p* = 0.750761)	1.187596 (*p* = 0.618021)
*Apoc2* Acc: NM_001085352.1 ID: 292697	Apolipoprotein C-II (RGD1560725)	2.057009↑ (*p* = 0.000592)	0.532413↓ (*p* = 0.000001)
*Apoc3* Acc: NM_012501.1 ID: 24207	Apolipoprotein C-III (ApoC-III, apo-CIII)	0.684698↓ (*p* = 0.000263)	1.307856↑ (*p* = 0.008469)
*Apoe* Acc: NM_138828.2 ID: 25728	Apolipoprotein E (APOEA)	1.485673↑ (*p* = 0.000003)	0.778654↓ (*p* = 0.004399)
*Apof* Acc: NM_001024351.1 ID: 500761	Apolipoprotein F	─	─
*Lpl* Acc: NM_012498.2 ID: 24539	Lipoprotein lipase	1.135422 (*p* = 0.100839)	0.812916↓ (*p* = 0.007915)
*Vldlr* Acc: NM_013155.2 ID: 25696	Very low density lipoprotein receptor	0.6722↓ (*p* = 0.000001) Or 0.486999↓ (*p* = 8.10E-08)	1.096751 (*p* = 0.283284) Or 1.520316↑ (*p* = 0.002103)
*Ppt1* Acc: NM_022502.2 ID: 29411	Palmitoyl-protein thioesterase 1 (Ppt)	0.9252 (*p* = 0.307699)	1.076592 (*p* = 0.320819) (*p* = 0.192733)
	Central Metabolic Regulators (Lipid Transcription Factors)		
*Foxa1* Acc: NM_012742.1 ID: 25098	Forkhead box A1	0.828962 (*p* = 0.418498)	1.067287 (*p* = 0.72547)
*Hnf4a* Acc: NM_022180.1 ID: 25735	Hepatocyte nuclear factor 4, alpha (Hnf4alpha, Hnf4a)	1.208695 (*p* = 0.660749)	0.934321 (*p* = 0.806661)
Mlxipl Acc: NM_133552.1 ID: 171078	MLX interacting protein-like (ChREBP, WS-bHLH, Wbscr14, bHLHd14)	1.019326 (*p* = 0.831873)	1.23554↑ (*p* = 0.015243)
*Nr1h3* Acc: NM_031627.2 ID: 58852	Nuclear receptor subfamily 1, group H, member 3 (LXRalpha)	0.898224 (*p* = 0.132662)	1.22274↑ (*p* = 0.00316)
*Ppara* Acc: NM_013196.1 ID: 25747	Peroxisome proliferator activated receptor alpha (PPAR)	1.042152 (*p* = 0.702728)	1.34318↑ (*p* = 0.01657)
*Ppard* Acc: NM_013141.2 ID: 25682	Peroxisome proliferator activated receptor delta (Pparb)	1.049635 (*p* = 0.640966) and 1.023475 (*p* = 0.858495)	1.001442 (*p* = 0.988966) and 0.929482 (*p* = 0.61664)
*Pparg* Acc: NM_013124.3 NM_001145366.1 NM_001145367.1 ID: 25664	Peroxisome proliferator activated receptor gamma	1.140175 (*p* = 0.07102)	0.96347 (*p* = 0.648174)
*Ppargc1a* Acc: NM_031347.1 ID: 83516	Peroxisome proliferator activated receptor gamma, coactivator 1 alpha (Ppargc1)	2.166136↑ (*p* = 0.013221)	0.49897 (*p* = 0.089406)
*Ppargc1b* Acc: NM_176075.2 ID: 291567	Peroxisome proliferator activated receptor, coactivator 1 beta (PGC1beta, Perc)	0.787663↓ (*p* = 0.007863)	1.092996 (*p* = 0.296335)
*Srebf1* Acc: XM_213329.5 XM_001075680.2 ID: 78968	Sterol regulatory element binding transcription factor 1 (ADD-1, ADD1, SREBP-1, SREBP-1c, Srebp1)	0.810661↓ (*p* = 0.012341) and 0.885992 (*p* = 0.449261)	1.17608 (*p* = 0.063087) and 1.460992↑ (*p* = 0.011985)
Srebf2 Acc: NM_001033694.1 ID: 300095	Sterol regulatory element binding transcription factor 2 (SREBP-2, SREBP2-retired, Srebf2)	0.854579 (*p* = 0.062966)	0.964258 (*p* = 0.651328)
*Xbp1* Acc: NM_001004210.1 ID: 289754	X-box binding protein 1 (HTF)	1.168979 (*p* = 0.087563)	1.080755 (*p* = 0.380509)

## Discussion

The objective of this study was to examine the effects of dietary administration of NDGA on gene expression involved in lipid homeostasis in liver, skeletal muscle and adipose tissue in rats chronically fed a 60 % fructose diet. A secondary goal was to utilize the information about the expression patterns of genes to identify lipid genes and pathways and modulators of lipid metabolism that might be the targets of NDGA amelioration of hypertriglyceridemia and hepatic steatosis. We examined gene expression in liver, gastrocnemius muscle (a representative of skeletal muscle) and white adipose tissue when rats were fed a chow, or HFrD supplemented with ± NDGA. We selected liver, skeletal muscle and adipose tissue for the following reasons: 1) the liver is a major organ responsible for fatty acid catabolism, de novo lipid synthesis (lipogenesis), triglyceride synthesis and export in the form of VLDL-TG; 2) skeletal muscle is a major site of fatty acid oxidation; and 3) fat stored as triglyceride in adipose tissue and mobilized in the form of plasma free fatty acids (FFAs) is the major fuel reserve and fuel source. In addition, all three tissues are highly sensitive to insulin action. We identified a number of genes involved in fatty acid oxidation, lipogenesis, and lipid clearance, as well as transcription factors influencing lipid metabolism, whose expression was significantly altered in response to NDGA treatment in these three tissue types.

As reported previously, and confirmed here, after 16 weeks on a diet in which about 67 % of kcals are derived from fructose, the rats developed hyperinsulinemia (insulin resistance), hypertriglyceridemia and hepatic steatosis [47, 56, 68]. Additionally, our results demonstrated that the dietary administration of NDGA greatly attenuated the HFrD induced hepatic steatosis and plasma triglyceride levels. Given that hepatic steatosis and dyslipidemia result from an imbalance in lipid homeostasis in the liver when fatty acid uptake or *de novo* lipogenesis outweighs lipid oxidation or export. Accordingly, we first examined the effects of HFrD and HFrD + NDGA feeding on the expression of key genes involved in hepatic fatty acid uptake, fatty acid oxidation and thioesterification/activation of fatty acids catalyzed by acyl-CoA synthetase enzymes, which is required for fatty acid catabolism, de novo lipogenesis and remodeling of biological membranes. Whereas expression of fatty acid transporter Slc27a1 (FATP1) was not affected by high-fructose feeding, expression of Slc27a2 (FATP2) was significantly upregulated. Likewise, although HFrD + NDGA treatment had no effect on the mRNA levels of Slc27a1, co-treatment with NDGA prevented the HFrD-induced expression of Slc27a2. We further observed that NDGA upregulated the expression of several genes for enzymes involved in fatty acid oxidation, including Cpt1b, Cpt2, Acox1, Acadvl, Eci and Ehbadh. In addition, our data provide evidence that NDGA differentially impacted the PPARα target genes for the enzymes involved in fatty acid thiolation/ activation (synthesis of acyl-CoAs); it increased the expression of acyl-CoA synthetase long-chain family member 1 (Acsl1) and acyl-CoA synthetase medium-chain family member 3 (Acsm3), but attenuated the levels of acyl-CoA synthetase long-chain family member 4 (Acsl4) and acyl-CoA synthetase short-chain family member 2 (Acss2). These acetyl-CoA synthetases vary in their substrate specificity and partitioning of fatty acids towards diverse cellular metabolic pathways. Acsl1shows preference for long chain saturated and monounsaturated fatty acids C16 to C18, whereas Acsl4 shows specificity for polyunsaturated fatty acids C20:4 and C20:5 [65]. Acsm3 acts on medium chain fatty acids C4 to C11 and catalytic function of Acss2 is to catalyze the activation of acetate (short chain) for use in lipid synthesis. Since C4-C18 fatty acids are mainly oxidized via mitochondrial β-oxidation, it is likely that NDGA induces the expression of Acsl1 and Acsm3 in an effort to promote increased channeling of the hepatic medium and long chain fatty acids for their catabolism via mitochondrial β-oxidation. Besides, we observed that NDGA treatment increased the gene expression of PPARα transcription factor, a member of the nuclear hormone receptor superfamily [60]. It is considered a master regulator of oxidative catabolism of fatty acids for energy production, is expressed at high levels in metabolically active tissues such as heart, liver, skeletal muscle and kidney, and controls the transcription of many genes involved in mitochondrial and peroxisomal β-oxidation and microsomal ω-oxidation of fatty acids, with liver being its major site of action [60, 64]. Some of the genes involved in fatty acid uptake and activation, such as SLC27A2, Acsl1, Acsl4, Acm3 and Acss2, are also the targets of PPARα [64].

In this study, NDGA also markedly increased the expression of both PPARα and PPARβ/δ in skeletal muscle, which was accompanied by increased expression of several genes for enzymes involved in fatty acid uptake, activation and oxidation, including Cpt1c, Acox1, Acadsb, Eci, Ehhadh, FATP2 (Slc27a2) and Acss2. Few genes were downregulated in response to NDGA treatment, including Cpt1a, Cpt1b, Eci2, Acsl2 and FATP1 (Slc27a1). It is unclear why NDGA treatment decreased the expression of three fatty acid catabolizing genes, Cpt1a, Cpt1b and Eci2. Also, it is surprising that NDGA treatment caused simultaneous mRNA induction of both PPARα and PPARβ/δ, given that in skeletal muscle, these PPARs show redundancy in their functions as regulators of fatty acid homeostasis [66]. Moreover, unlike in the liver and heart, PPARβ/δ is several-fold more abundant in skeletal muscle than PPARα [62, 63, 66, 67, 69] and that the β/δ-sub-type can compensate for deficiency of PPARα in this tissue [66]. In addition, the metabolic significance of NDGA induction of skeletal muscle PPARα is not apparent given that PPARα gain-of-function and loss-of-function studies in mice have shown that the activity of the skeletal muscle PPARα pathway is directly linked to the development of insulin resistance, glucose intolerance, and diabetes [70]. More specifically, it has been said that overexpression of PPARα in skeletal muscle increases the expression of genes for fatty acid uptake and oxidation, protects mice against diet-induced obesity, but decreases the expression of the glucose transporter Glut4, resulting in animals becoming insulin resistant and glucose intolerant [70]. In contrast, diet-induced insulin resistance was prevented in PPARα-/- mice despite the development of obesity. Our previous studies, however, have shown that treatment of high-fat diet fed mice [59] and high-fructose diet fed rats [68] with NDGA improves whole body insulin resistance and glucose intolerance. Obviously, more experimental evidence will be needed to sort out the role of PPARα in skeletal muscle. Similar to liver and skeletal muscle, NDGA treatment also increased the expression of PPARα in white adipose tissue (WAT); however, it did not significantly impact the expression of genes for enzymes/proteins involved in fatty acid uptake and oxidation. The lack of effect of NDGA on fatty acid oxidative genes in adipose tissue is likely due to the fact that the fatty acid storage function of adipose tissue-specific PPARγ2 predominates over that of the oxidative function of PPARα [71, 72].

A reduction in hepatic lipogenesis appears to be an alternative mechanism for the lipid-lowering effect of NDGA. While high-fructose feeding increased the expression of sterol regulatory element-binding protein-1c (SREBP-1c) [73, 74] and carbohydrate responsive element-binding protein (ChREBP or Mlxipl) [74, 75], the two transcription factors that primarily regulate hepatic de novo lipogenesis (DNL), NDGA treatment prevented the upregulation of these transcription factors. The nutrition-sensitive SREBP-1c regulates the expression of hepatic lipogenic enzymes (FAS, ACL, ME, S_14_, SCD1, GPAT1, FADS1, FADS2, ELOVL5, ELOVL6, MTTP, G6PD) and glycolytic enzymes (GCK, PKLR) [73,74] in response to insulin [75, 76], whereas ChREBP regulates some of these genes (L-PK, SREBP-1c, ACL, FAS, ACC, S14, DGAT2, ME, ELOVL2, GLUT4, GCKR) [77, 78] in response to high glucose [76, 79]. NDGA treatment markedly reduced the expression of a large number of SREBP-1c and/or ChREBP target genes including *Acly*, *Fasn*, *Scd1*, *Elovl2*, *Elovl5*, *Fads1*, *Fads2*, *DGAT2* and *Pklr* as compared to rats fed a high-fructose diet alone. Surprisingly, NDGA treatment also increased the gene expression of a few genes, such as *Elovl4*, *Elovl6*, and *Mogat1*. The reason for their upregulation is not clear, but may be related to the fact that these genes, along with several other lipogenic genes, are also regulated by PPARα [64], whose expression, as noted above, is upregulated in response to NDGA treatment. NDGA also increased (*Lpl*, *Vldr*) and decreased (*Apoa4*, *Apof*) the expression of genes involved in lipid clearance. Again, *Lpl* and *Vldr* are known targets of PPARα [64]. Interestingly, expression of hepatic glycolytic genes, *Gck* and *Gckr*, was decreased by NDGA treatment; glucokinase (GCK) in liver and pancreatic β-cells is subject to inhibition by glucose regulatory protein (GCKR) [80, 81]. The inhibitory effect of GCKR depends on the presence of fructose-6-phosphate (F6P) and is antagonized by fructose-1-phosphate (F1P) [80, 81]. The NDGA inhibition of *Gck* is likely to contribute to NDGA-mediated attenuation of lipogenesis, since GCK is the rate-limiting enzyme of glycolysis in the liver, and stimulates glycolysis and lipogenesis by enhancing glucose flux, including production of acetyl-CoA for lipid synthesis [82]. The simultaneous inhibition of expression of both *Gck* and *Gckr* in response to NDGA could be explained on the basis of NDGA-mediated inhibition of SREBP-1c and ChREBP; *Gck* is a known target of SREBP-1c [73, 74], whereas *Gckr* is regulated by ChREBP [77]. In addition, NDGA-inhibition of *Gck* may further limit lipogenesis, given that GCK also stimulates glycolysis and lipogenesis at the transcription level via ChREBP [79].

In addition, we found that NDGA increased the gene expression of malonyl decarboxylase (Mlycd) enzyme, which catalyzes the degradation of malonyl-CoA to acetyl-CoA [83]. Therefore, it is likely that the availability of malonyl-CoA as a substrate for lipogenesis is substantially lower in the livers of NDGA treated animals, and, thus, limits the extent of lipogenesis. Apart from its role as the immediate precursor for *de novo* synthesis of fatty acids, malonyl-CoA plays an important role in regulating hepatic fatty acid oxidation as a potent inhibitor of carnitine palmitoyitransferase [84, 85]. Therefore, NDGA-dependent increases in Mlycd expression and subsequently decreases in malonyl-CoA levels may stimulate mitochondrial transport of fatty acids for their mitochondrial β-oxidation. Interestingly, Mlycd is a potent target of PPARα [64, 86, 87].

Similar to the liver, NDGA treatment differentially impacted the genes for enzymes/proteins involved in *de novo* lipogenesis, elongation and desaturation of fatty acids, triglyceride synthesis and lipid clearance in skeletal muscle and adipose tissue. These changes, as discussed above for the liver, are likely to result from interplay between PPARα, PPARβ/δ, PPARγ, SREBP-1c, and ChREBP in a skeletal muscle and adipose tissue specific manner. Surprisingly, no NDGA-induced changes in Mlycd expression were noted in either skeletal muscle or adipose tissue. Other studies have previously reported significant changes in Mlycd levels in both tissues under variable physiological conditions [88, 89]. Since Mlcyd is also regulated by AMPK by a phosphorylation/dephosphorylation mechanism, it is possible that NDGA may stimulate enzymatic activity by promoting its AMPK-mediated phosphorylation [89]. This is a likely possibility given that NDGA treatment causes a robust stimulation of AMPK activity via its increased phosphorylation.

## Conclusions

In conclusion, our study shows that chronic dietary treatment with NDGA can attenuate high-fructose diet-induced hypertriglyceridemia and hepatic steatosis (TG accumulation). DNA microarray analysis showed that dietary NDGA profoundly affects the gene expression profiles in liver and skeletal muscle. However, adipose tissue was less dramatically impacted by NDGA treatment. Analysis indicated that NDGA differentially increased the expression of enzymes involved in hepatic fatty acid oxidation and of proteins that facilitate fatty acid transport. The changes were generally greater in liver and skeletal muscle than in adipose tissue. The high-fructose diet containing NDGA also altered the expression of genes related to proteins/enzymes of de novo fatty acid and triglyceride synthesis and lipid clearance. On the basis of the experimental data obtained, the beneficial actions of NDGA on hypertriglyceridemia and steatosis are exerted by a dual mechanism: inhibition of lipogenesis and enhanced functional expression of the key genes for enzymes/proteins involved in fatty acid β-oxidation pathway in liver and skeletal muscle. In adipose tissue, although NDGA inhibits the expression of major enzymes involved in lipogenesis, it also promotes lipid storage by upregulating the expression of TG synthesizing enzymes and attenuating the mRNA levels of proteins that catalyze TG hydrolysis.

## Methods

### Materials

Oil Red O was obtained from Sigma-Aldrich (St. Louis, MO). Glucose, TG, FFA, and cholesterol measurement kits were obtained from Stanbio Laboratory (Boerne, TX). The rat insulin RIA Kit was obtained from EMD Millipore (Billerica, CA). 60 % Fructose diet (high fructose diet [HFrD] #TD.89247; formula g/Kg: Casein 207; DL-Methionine 3.0; Fructose 600.0; Lard 50.0; Cellulose79.81; Mineral Mix, Rogers-Harper [170760] 50.0; Zinc Carbonate 0.04; Vitamin Mix, Teklad [40060] 10.0 and Green Color 0.15; percent by weight [% kcal from]: Protein 18.3 [20.2]; Carbohydrate 60.4 [66.8]; and Fat 5.2 [13.0]) from Envigo Teklad Diets, Madison, WI. 60 % Fructose diet supplemented with NDGA (2.5 NDGA/kg HFrD) was also custom made by Envigo Teklad Diets. All other reagents used were of analytical grade.

### Animals and treatments

All animal experiments were performed according to the procedures approved by the VA Palo Alto Health Care System Animal Care and Use Committee (IACUC). Male Sprague-Dawley (SD) rats, obtained from Harlan Sprague-Dawley (Indianapolis, IN), were used in these studies. For dietary treatment of rats with NDGA, groups of SD rats were fed a standard chow diet for 1 week to acclimatize them to a new environment. Subsequently, one group of rats switched to HFrD supplemented with 2.5 g NDGA/g HFrD and was maintained on this diet for 16 weeks. The second group of SD rats was maintained on HFrD alone for 16 weeks, while a third group of rats continued to be fed a chow diet for 16 weeks. Before, during, and at the end of the feeding period, blood samples were taken from the tails of rats for analyses of serum metabolites after 4 h of fast. At the end of the 16-wk treatment period, the rats were fasted, blood was collected, and rats were subsequently euthanized. Blood samples were centrifuged at 4,000 x *g* for 15 min at 4 °C and the recovered serum samples were stored at –80 °C until analyzed for various metabolites. Liver, skeletal muscle (mixed gastrocnemius; ~3 % type I fiber, ~6 % type IIA fiber, ~34 % type IID/X fiber and ~57 % type IIB fiber) [90] and white adipose tissue (WAT, epididymal) were excised immediately, weighed, frozen in liquid nitrogen, and stored at –80^o^C until utilized for gene array analysis, the quantification of liver TG content or Oil Red O staining of frozen sections for steatosis evaluation.

### Measurement of serum triglyceride, cholesterol, glucose, free-fatty acids and insulin levels

Serum glucose, triglyceride, free-fatty acids and total cholesterol levels were determined with commercial assay kits (Stanbio Laboratory, Boerne, TX). Serum insulin levels were quantified using a rat specific insulin RIA Kit (EMD Millipore, Billerica, CA).

### Quantification of hepatic triglyceride content

Suitable aliquots of liver tissue homogenates were extracted with chloroform-methanol according to the procedure of Folch et al [91], and extracted lipid samples were analyzed for their TG content with an enzymatic assay kit as noted above.

### Whole genome microarray

RNA samples for the whole genome microarray (Rat OneArray^®^ v1) were isolated from the liver, white adipose tissue (epididymal) and skeletal muscle (gastrocnemius) using Qiagen RNeasy Plus Mini Kit (Valencia, CA). The RNA analysis, including integrity and quantitation of RNA, was carried out using an Agilent RNA 6000 Nano Kit and an Agilent Bioanalyzer System (Agilent Technologies, Santa Clara, CA). Total RNA was pooled in an equal amount from individual rats of each group and treated with DNase I to remove residual genomic DNA. cDNA synthesis, Cy5-labelling and hybridization to the GeneChip GPL 13694 (rat OneArray^®^ v1) were performed by PhalanxBio, Inc. (San Diego, CA). This assay is designed to generate amplified and Cy5 sense-strand DNA targets from the entire expressed genome without bias. Three repeats from each group were performed. The microarray data files have been submitted to the Gene Expression Omnibus; the accession number is GSE81346 (http://www.ncbi.nlm.nih.gov/geo/query/acc.cgi?acc=GSE81346).

### Differential and cluster gene expression analysis

The raw data from microarrays were analyzed using Partek Genomics Suite software, version 6.3 copyright 2008 (Partek Inc., St. Louis, MO). Briefly, GenePix Results (GPR) files containing the hybridization intensity were imported, followed by quintile normalization and log2 transformation to represent gene expression levels. Liver samples were grouped into: 1) standard chow diet fed (control); 2) high-fructose diet (HFrD)-fed; and 3) HFrD-NDGA fed animals. Three-way ANOVA was performed including diet (chow and HFrD) and NDGA interaction to generate the lists of differentially expressed genes comparing chow, HFrD and HFrD-NDGA. There were three microarrays for each group. For the comparison between chow-fed and HFrD-fed rats, probe sets with a fold change of 2.0 and adjusted *P* value <0.05 were considered differentially expressed. For the comparison between the HFrD and HFrD-NDGA groups, the analyses were also set with a fold change of 2.0 and adjusted *P* value of <0.05. The Benjamini-Hochberg false discovery rate (FDR) method was used to select the regulated genes with the lowest FDR. Partek Genomics Suite was used as the first step for quality control of the data on all the samples with two methods: Pearson correlation and Principal Component Analysis (PCA). PCA was performed as a global view of sample clustering, which is related to the total variance in gene expression for all genes. Normalized expression values for all genes were analyzed.

### Pathway analysis

For each comparison, a list of differentially expressed genes was generated. The gene list, along with associated expression or fold-change values, were further analyzed using Ingenuity Pathway Analysis (Ingenuity Systems, Inc., Redwood City, CA) to identify differentially expressed pathways induced by HFrD feeding or NDGA in HFrD rats. The list of significantly regulated genes, selected by the microarray analysis described above, was loaded in IPA with the following criteria: direct and indirect relationships filtered by species (rat) and by tissue (liver, gastrocnemius or adipose tissue). Then IPA computed the data to generate significant networks of genes that are associated with particular biological functions, diseases and metabolic/signaling pathways.

### Oil Red O staining of liver sections for detection of neutral lipids

Liver samples were collected from rats, embedded in Tissue Freezing Medium (Leica Microsystems Inc., Buffalo Grove, IL), and stored at -80 °C until used for sectioning to 8-μM slices. Liver sections were stained with Oil Red O for the visualization of neutral lipids stored in the lipid droplets by using a slight modification of the standard procedure as described earlier from this laboratory (59).

### Statistical analysis

All of the data are expressed as the means ± S.E. One way and two-way ANOVA were used to study differences among groups. This was followed by a post hoc comparison using Bonferroni’s multiple-comparison test when necessary. Differences at *P* < 0.05 were considered statistically significant. Prism 5 software (GraphPad, La Jolla, CA) was used for all of the statistical calculations.

## Abbreviations

FC: Free cholesterol
FFAs: Free fatty acids
HFrD: High-fructose rich diet
MetS: Metabolic syndrome
NDGA: Nordihydroguaiaretic acid
NAFLD: Non-alcoholic fatty liver disease
NASH: Nonalcoholic steatohepatitis
TC: Total cholesterol
TG: Triglyceride
